# A common garden experiment supports a genetic component underlying the increased resilience of common cockle (*Cerastoderma edule*) to the parasite *Marteilia cochillia*

**DOI:** 10.1111/eva.13601

**Published:** 2023-10-17

**Authors:** Antonio Villalba, Raquel M. Coimbra, Marina Pampín, David Iglesias, Damián Costas, Carlos Mariño, Andrés Blanco, Manuel Vera, Marta Domínguez, Eva Cacabelos, Emilio Abella, Mónica Incera, Rosa Fernández Otero, Paulino Martínez

**Affiliations:** ^1^ Centro de Investigacións Mariñas (CIMA), Consellería do Mar, Xunta de Galicia Vilanova de Arousa Spain; ^2^ Departamento de Ciencias de la Vida Universidad de Alcalá Alcalá de Henares Spain; ^3^ Research Centre for Experimental Marine Biology and Biotechnology (PIE) University of the Basque Country (UPV/EHU) Plentzia Spain; ^4^ Departamento de Pesca e Aquicultura Universidade Federal Rural de Pernambuco Recife Brazil; ^5^ Departamento de Zoología, Genética y Antropología Física, Facultad de Veterinaria, Campus Terra Universidade de Santiago de Compostela Lugo Spain; ^6^ Centro de Investigación Mariña Universidade de Vigo, ECIMAT Vigo Spain; ^7^ Confraría de Pescadores S. Antonio Cambados Spain; ^8^ Hydrosphere S. L. Vigo Spain; ^9^ Instituto de Investigaciones Marinas, Consejo Superior de Investigaciones Científicas IIM‐CSIC Vigo Spain; ^10^ Confraría de Pescadores A Pastoriza Vilanova de Arousa Spain; ^11^ Centro Tecnolóxico do Mar ‐ Fundación CETMAR Vigo Spain

**Keywords:** bivalve mollusk, candidate genes, genetic differentiation, natural selection, single nucleotide polymorphism

## Abstract

The common cockle is a valuable bivalve species inhabiting the Atlantic European coasts. The parasite *Marteilia cochillia* has devastated cockle beds in the southern Galician (NW Spain) rias since 2012. Previous data suggested that cockles from Ría de Arousa acquired some resilience to this parasite through natural selection after consecutive annual marteiliosis outbreaks and candidate markers associated with marteiliosis resilience were identified using population genomics and transcriptomics approaches. Here, a common garden experiment was performed using a naïve stock (from Ría de Muros‐Noia) and an affected stock (from Ría de Arousa) to test this hypothesis. Breeders from both stocks were used to produce seed cohorts at hatchery, which were pre‐grown in a raft (outdoor nursery stage) and deployed in two shellfish beds affected by marteiliosis in Ría de Arousa (growing‐out stage). In both beds, the naïve stock showed high marteiliosis prevalence and was fully depleted in a short period, while the affected stock barely showed evidence of marteiliosis. A set of 45 SNPs putatively associated with marteiliosis resilience were fitted for MassARRAY genotyping to check their role in the differential resilience detected between both stocks. Though no significant differentiation was found between the naïve and the affected stocks with neutral markers, 28 SNPs showed significant divergence between them, suggesting that these SNPs were involved in directional selection during eight generations (to the most) of marteiliosis pressure (long‐term selection). Furthermore, signals of selection were also detected in the naïve stock along the marteiliosis outbreak in the growing‐out stage (short‐term selection) and six SNPs, all shared with the long‐term evaluation, showed consistent signals of differentiation according to the infection severity. Some of these SNPs were located within immune genes pertaining to families such as proteasome, ubiquitin, tumor necrosis factor, and glutathione S‐transferase. These resilience‐associated markers will be useful to recover cockle production in Galicia.

## 1 INTRODUCTION

The common cockle *Cerastoderma edule* (Linnaeus 1758) is a marine bivalve occupying intertidal and shallow subtidal sediments in the Northeast Atlantic coast extending from Norway and Iceland down to the Northwestern coast of Africa (Krakau et al., 2012). It is the main wild‐harvested bivalve species in Europe, annually accounting for as much as 18,000 tons and playing an essential role in ecosystem services (Carss et al., 2020). Cockle harvesting in Galicia (NW Spain) is an important income source. It is performed from boat (subtidal beds) or on foot (intertidal beds), the latter conducted primarily by women (>95%) (Piñeiro‐Antelo & Santos, 2021).

The production of *C. edule* varies across its distribution range and seasons due to different causes such as diseases, overharvesting, climate variations, and water waste treatment (Braga et al., 2022; Burdon et al., 2014; Callaway, 2022; Carballal et al., 2001; Crespo et al., 2010; Longshaw & Malham, 2013). The largest cockle harvesting in Spain occurs in Galician rias (the so‐called coastal inlets formed by the partial submergence of an unglaciated river valley). A disease outbreak was detected in February 2012 in Ría de Arousa (NW Spain), the ria with the highest common cockle production, leading to the cockle fishery collapse. The protistan parasite *Marteilia cochillia* (Carrasco et al., 2013) was identified as the main cause, reaching 100% prevalence in just over 2 months (Villalba et al., 2014). This parasite proliferates through the cockle digestive gland, where food absorption mostly takes place, up to tightly occupy all the absorptive epithelium of the digestive gland tubules, which become atrophied when parasite cells are massively shed into digestive lumina at the last infection stage, thus causing dysfunction of the food absorptive organ (Iglesias et al., 2023). Infection by *M. cochillia* produces emaciation, discoloration of the digestive gland, cessation of growth, and acute cockle mortalities (Carrasco et al., 2013; Villalba et al., 2014). The common cockle's average density in exploited beds located in the Ría de Arousa used to be 210 ind/m^2^ between 2002 and 2012, but after the marteiliosis outbreak, it decreased to 0 ind/m^2^ in August 2012, especially in the innermost area, where the Lombos do Ulla shellfish bed is located (Villalba et al., 2014). Later, between 2013 and 2014, the disease spread to southern rias, namely, Ría de Pontevedra and Ría de Vigo, but interestingly, no outbreak has been detected in the northward adjacent Ría de Muros‐Noia, less than 30 km away from Ría de Arousa (Iglesias et al., 2017, 2023).

A previous study of marteiliosis dynamics in Ría de Arousa from 2012 to 2019 showed that the cumulative mortality and marteiliosis prevalence in cockle cohorts recruited since 2016 in Lombos do Ulla were much lower than in the period 2012–2015, suggesting that resilience to the parasite could have increased by natural selection (Iglesias et al., 2023). Consistently, naïve cockles from the shellfish bed of Noia (Ría de Muros‐Noia), where marteiliosis outbreaks have never been detected, that were transplanted into the heavily affected shellfish bed Lombos do Ulla (Ría de Arousa) in 2017 and 2018 showed much higher cumulative mortality and marteiliosis prevalence than cockles naturally recruited to Lombos do Ulla (Iglesias et al., in press).

To ascertain the genetic variation underlying complex traits, highly influenced by environmental factors, it is advisable to homogenize the environment to ensure that phenotypic differences observed among the analyzed samples or strains are due to genetic differences, and this can be attained by using common garden strategies (de Villemereuil et al., 2016). These experiments can be conducted in a more controlled environment at indoor facilities, or in the wild, where environmental variation is greater and less predictable. In the case of marteiliosis¸ a presumable complex trait according to previous data (Pampín et al., 2023), infection at indoor facilities is not feasible because direct transmission from infected cockles to healthy ones does not seem possible given the complex life cycle postulated for *Marteilia* spp., involving at least one intermediate host (Berthe et al., 2004; Carrasco et al., 2015; Iglesias et al., 2023). Furthermore, procedures for in vitro culture of *Marteilia* spp. (including *M. cochillia*) have not been established yet. Thus, a common garden experiment in a natural environment, where besides the cockle production takes place, would be the most appropriate approach since all relevant exposure routes are likely operating. Checking for the genetic basis of disease resilience in other bivalve species has been carried out through common garden approaches in the wild (Casas et al., 2017; da Silva et al., 2005; Dégremont et al., 2019; Dove et al., 2013; Farhat et al., 2020), whereas availability of large quantity of pathogens allowed performing indoor common garden experiments (Dégremont et al., 2021; Yang et al., 2022; Zhao et al., 2021).

A previous genome scan in common cockle identified a set of 233 candidate SNP markers associated with resilience to marteiliosis using population genomics and transcriptomics approaches in samples with different degree of infection or exposure status collected in Ría de Arousa (Pampín et al., 2023). A total of 45 SNPs were selected as the most confident markers: 12 showing signals of divergent selection from 9154 2b‐RAD genotyped SNPs (Vera et al., 2022), and 33 associated with differentially expressed genes (DEG) through RNAseq from the enriched digestive gland transcriptome (Pardo et al., 2022). However, their application for disease management and breeding programs requires their technical validation as well as checking their ability to discriminate resilient and susceptible phenotypes.

Pampín et al. (2023) suggested that these candidate SNPs could underlie marteiliosis resilience and to test this hypothesis, we designed a common garden experiment in Ría de Arousa, where cockle marteiliosis occurs, using a naïve cockle stock and a cockle stock affected by marteiliosis over several generations. The specific objectives of our study were (1) to confirm that marteiliosis resilience of cockles increased through natural selection after several outbreaks and (2) to assess if allelic variants of candidate SNPs could explain the resilience to the parasite. This information could be useful to discriminate naïve and resilient cockles for their eventual application in Marker‐Assisted Selection (MAS) programs and for management of wild resources of common cockle in Galician rias.

## 2 MATERIALS AND METHODS

### 2.1 Common garden experiment

**FIGURE 1:**
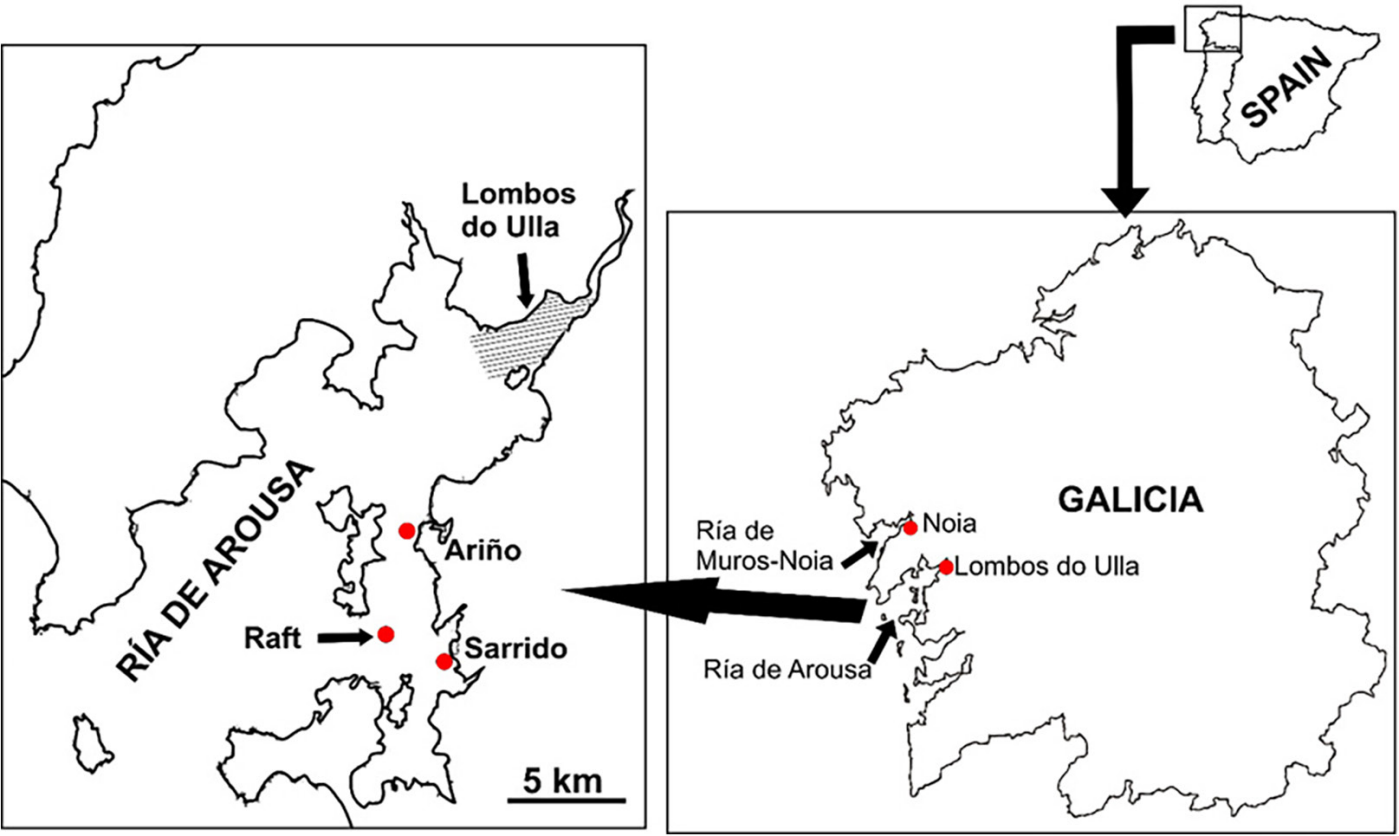
Zoomed mapping showing (right) the geographical origin of the two *Cerastoderma edule* stocks, Noia (Ría de Muros‐Noia, naïve) and Lombos do Ulla (Ría de Arousa, affected), in Galicia (NW Spain). Left: map of Ría de Arousa showing the location (red spots) of the raft (outdoor nursery stage) and the two shellfish beds, Ariño and Sarrido, where the growing‐out stage of the common garden experiment took place.

The common garden experiment involved three stages in which cockles of two stocks shared the same environment: (1) seed production at hatchery, (2) outdoor nursery stage on a culture raft, and (3) growing‐out stage in two shellfish beds. Adult cockles to be used as breeders to produce seed cohorts at hatchery were harvested in two shellfish beds (400 individuals per bed), one of them in Ría de Arousa, which has been heavily affected by marteiliosis since its first detection in 2012 (Lombos do Ulla, affected stock), and the other one in Ría de Muros‐Noia, where no marteiliosis outbreak has ever been detected (Noia, naïve stock). Cockles were collected from Lombos do Ulla in late May 2020 and from Noia in early June 2020 (Figure 1), within the period of ripe‐spawning gonad of cockles in both shellfish beds (Iglesias, 2006). Subsequently, the cockles were transferred to the hatchery facilities of the *Estación de Ciencias Marinas de Toralla* (ECIMAT, *Centro de Investigación Marina, Universidad de Vigo*) and distributed into tanks (100 cockles per tank) with pumped filtered seawater plus cultured phytoplankton for acclimation. Although we cannot discard that some breeders coming from Lombos do Ulla might be infected, it does not appear that vertical and horizontal transmission of *Marteilia* among cockles by cohabitation in tanks is possible. This is supported by repeated failed attempts to transmit the disease from infected to healthy cockles by cohabitation in tanks (Villalba et al., unpublished results) and further considering the complex life cycle postulated for *Marteilia* spp. involving at least one intermediate host (Berthe et al., 2004; Carrasco et al., 2015; Iglesias et al., 2023). Spontaneous or temperature‐induced spawning took place in the tanks in the following days. Several larval cohorts were produced from Noia and from Lombos do Ulla breeders, which were separately reared in tanks for approximately 70 days in the hatchery (until 27th August 2020). After this period, all the seed cohorts from each stock were pooled, and distributed into plastic mesh bags, which were set in perforated trays to be transferred to a raft in the Ría de Arousa for the outdoor nursery stage until 13th November 2020 (Figure 1; Figure S1). Throughout this phase, cleaning and density readjustments were performed twice (on 17th September and 13th October). At the end of this period, the mean length (±SD) of the seeds was 16.8 ± 0.12 mm and 18.1 ± 0.13 mm for the Noia and the Lombos do Ulla stocks, respectively.

Seeds were taken from the raft and labelled with a blue dot (Noia stock) or a black dot (Lombos do Ulla stock) with a pen marker on the two shell valves, and the marks were covered with nail lacquer to prevent erasing. In November 2020, a total of 600 labeled cockles of each stock were transferred for the growing‐out stage in two intertidal shellfish beds of the Ría de Arousa, namely, Ariño and Sarrido (Figure 1), both affected by marteiliosis, at the time when the annual outbreak was expected to occur (Iglesias et al., 2023).

Twelve sets of 100 cockle seeds were respectively placed in 12 perforated plastic boxes (57 cm in length × 36 cm in width × 23 cm in height; three boxes of the naïve stock and other three of the affected stock in each shellfish bed), which were filled with sediment taken from the shellfish beds. To facilitate mortality evaluation, 15 cockles of each box were placed within a 17 cm side square enclosure made of plastic net in one corner of the box. The trays, covered with a plastic net of 1‐cm mesh to prevent predation, were subsequently buried in the sediment at the intertidal zone (Figure S2). Monitoring of cockles in each shellfish bed was planned to be performed until mid‐summer 2021, when average cockle size was expected to be around the minimum market size (25 mm in length). However, due to the high mortality observed in the Noia stock, monitoring had to be finished several months earlier in both shellfish beds. The Lombos do Ulla stock in the shellfish bed of Ariño also suffered high mortality anticipating the end of monitoring to April, whereas cockles from Lombos do Ulla stock deployed in the bed of Sarrido suffered much lower mortality and, thus, the monitoring period was covered as initially planned.

### 2.2 Monitoring of cockle seed from both stocks and shellfish beds

Mortality of the seed of each stock was estimated monthly since their deployment in the shellfish beds of Ariño and Sarrido. For this purpose, live and dead cockles in the corner enclosure of each box were counted and, whenever mortality was detected, live seeds from the remaining external area in the box were moved into the corner enclosure to maintain a constant number of 15 live seeds at the beginning of each inter‐sampling period. The cumulative mortality for each stock at each time in the two shellfish beds was estimated from the mortality rate until the monitoring of each stock was finished.

Cockle samples were also taken throughout the period of the experiment for marteiliosis diagnosis and for genotyping of markers associated with marteiliosis resilience. An initial sample was taken at the transference from the raft to the shellfish beds and, from then, cockles were collected every 2 months until the end of the experiment (25–57 cockles per sample; Table 1). The collected cockles were carried to the laboratory and kept in a tank with open seawater flow for 24 h to allow the elimination of gut contents. A piece of the gills from each cockle was preserved in absolute ethanol for DNA extraction and genotyping. Additionally, a transversal section (about 5 mm thick) of soft tissues, including gills, visceral mass, mantle, and foot, was taken and fixed in Davidson's solution for 24 h at 4°C, and then preserved in 70% ethanol until further histological processing. The fixed soft tissues were embedded in paraffin and sectioned (5 μm thick) with a rotary microtome. Sections were stained with Harris' hematoxylin and eosin (Howard et al., 2004). A histological section of each cockle was examined under light microscopy for marteiliosis diagnosis, using the scale proposed by Iglesias et al. (2023) to rank its severity: stage 0, when the parasite was not detected; stage 1, early infection; stage 2, moderate infection; stage 3, heavy infection; and stage 4, terminal stage after releasement of most parasites. Prevalence of marteiliosis was calculated as the percentage of the total number of infected individuals over the total sampled.

**TABLE 1:** Samples collected for diagnosis of marteiliosis and genetic analyses in *Cerastoderma edule* from the common garden experiment conducted in Ría de Arousa (Galicia, NW Spain).

Stock	Sampling site	Sampling date	No. cockles analyzed for diagnosis	No. cockles analyzed for SNP genotyping	Sample code (SNP genotyping)
Lombos do Ulla	Raft^a^	11/13/2020	50	34	L
	Sarrido	01/14/2021	30	30	LS1
		03/16/2021	30		
		05/11/2021	50	35	LS2
		07/12/2021	57		
	Ariño	01/13/2021	50	30	LA1
		03/15/2021	50	34	LA2
		04/13/2021	47	34	LA3
Noia	Raft^a^	11/13/2020	50	34	N
	Sarrido	01/14/2021	30	30	NS1
		03/16/2021	30	30	NS2
		04/14/2021	25	25	NS3
	Ariño	01/13/2021	30	30	NA1
		03/15/2021	45	34	NA2
Total Noia			210	183	
Total Lombos do Ulla			364	197	
Grand total			574	380	

*Note*: Stock codes: Lombos do Ulla (L) or Noia (N); shellfish bed: Sarrido (S) or Ariño (A).

^a^ Sampling was performed at the transference of cockle seed from the raft to the shellfish beds.

### 2.3 Candidate marker genotyping

DNA extraction from preserved gill pieces was performed with the DNeasy kit (QIAGEN). Samples were genotyped with a panel of 45 SNPs selected from a set of 233 SNPs putatively associated with marteiliosis resilience previously identified using population genomics (110) and transcriptomic (123) approaches (Pampín et al., 2023) (Table S1). SNP selection was done using both functional information (within or close to DEG related to immunity), discrimination power between naïve and affected samples, population genetics criteria (expected heterozygosity, conformance to Hardy–Weinberg equilibrium and missing data), and technical criteria (suitable flanking regions for primer design).

Primers for the MassARRAY genotyping were chosen by their technical feasibility to be combined in two multiplex reactions, taking as reference the cockle genome assembly (Bruzos et al., in press) (Table S1). Briefly, the technique consists of a two‐step reaction: the first involves the PCR amplification of an amplicon which includes the selected SNP (i.e., locus‐specific PCR reaction), and the second a single‐base extension using mass‐modified dideoxynucleotide terminators of an oligonucleotide primer that anneals immediately upstream of the SNP of interest (Ellis & Ong, 2017; Oeth et al., 2009). After in silico primer design, a panel of 45 SNPs was divided into multiplex 1 (25 SNPs) and multiplex 2 (20 SNPs) (see Table S1 for detailed information). MALDI‐TOF mass spectrometry analysis in an Autoflex spectrometer was used for allele scoring. Genotyping was conducted at the UCIM‐University of Valencia Genomics Platform.

### 2.4 Genetic diversity and structure

Genetic diversity per sample was estimated using average observed (*H*_O_) and expected (*H*_E_) heterozygosities across loci, and allelic richness using the R package diveRsity (1.9.90) (Keenan et al., 2013). The same program was used to check for conformance to Hardy–Weinberg Equilibrium (HWE) and intrapopulation fixation index (*F*_IS_). These parameters were calculated in all the samples collected from the initial transfer from the raft and along the growing‐out stage, considering both the stock (naïve—Noia and affected—Lombos do Ulla) and shellfish bed (Ariño and Sarrido). Pairwise Wilcoxon rank paired tests were performed to check for differences in genetic diversity estimators between and within stocks and shellfish beds. Wright's coefficient of genetic differentiation (*F*_ST_) was calculated per locus and across loci for all samples (global) and between all sample pairs (pairwise) considering the two stocks (Noia and Lombos do Ulla) and shellfish beds (Sarrido and Ariño) using the software ARLEQUIN v.3.5 (Excoffier & Lischer, 2010). Analyses of molecular variance (AMOVA) were performed to evaluate the distribution of genetic diversity between stocks (*F*_CT_) and among samples within stock (*F*_SC_) with ARLEQUIN v.3.5. Significance of genetic differentiation was calculated with ARLEQUIN using 10,000 permutations.

To estimate the number of population units (*K*), a Bayesian clustering method implemented in STRUCTURE (Pritchard et al., 2000) was performed with an admixture model with two populations, a burn‐in period of 10,000 and 1,00,000 Markov Chain Monte‐Carlo steps (MCMC). We tested from *K* = 1–13 considering the number of samples plus 1, and five independent replicate runs per test. The most likely number of *K* was estimated with the web‐based software StructureSelector (Li & Liu, 2018) and outputs were obtained with CLUMPAK (Kopelman et al., 2015). Additionally, complementary Discriminant Analysis of Principal Component (DAPC) evaluations were performed through the R package (Jombart, 2008; Jombart et al., 2010). First, a cross‐validation, function “xvalDapc” with 1000 replicates was performed to estimate the number of optimal principal components (PC) to be retained and used in the Linear Discriminant Analysis (LDA). The best number of PCs to be retained was performed with the lowest root mean square error (RMSE) approach and then data were transformed using principal component analysis (PCA). We also repeated the analyses with an appropriate number of principal components (PC) and discriminant functions (DF) to explain >90% of the variance. The resultant clusters were represented in a 2D scatterplot using the two best linear components of DAPC and the R function (“scatter”).

To explore the relationship among the different variables (stocks, infection status, and markers), multiple correspondence analyses (MCA) were performed with the function “dudi.mca” of the R package ade4 (Dray & Dufour, 2007; Thioulouse et al., 2018) considering two scenarios: (i) the whole sample collection (including both stocks, Lombos do Ulla and Noia), and (ii) the Noia stock samples. For these analyses, we excluded monomorphic loci and those with missing genotyping data >20%, since missing data can seriously hamper obtaining consistent results. Also, the infection status was simplified by pooling infected samples (infection.1: included 1–4 infection level) versus non‐infected (infection.0) to facilitate the visualization of the high number of variables handled. The two first dimensions explaining the highest variance component were represented in 2D plots, where each variable was included at its highest confident position. This information enabled to establish the relationship between stocks and infection status, but also to ascertain the relevance of each marker and genotype relative to those variables.

## 3 RESULTS

### 3.1 Mortality and marteiliosis diagnosis

**FIGURE 2:**
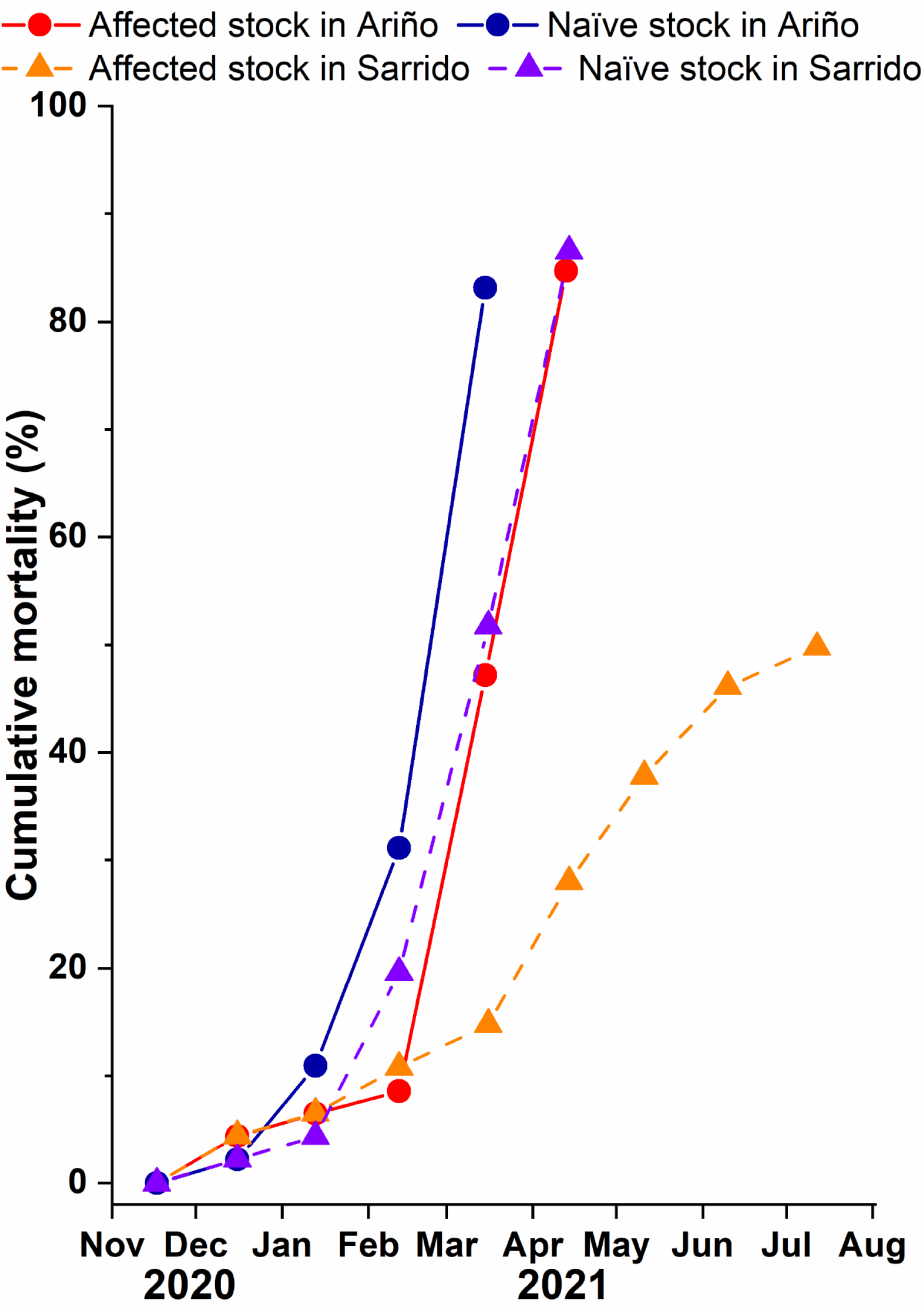
Cumulative mortality of cockles (*Cerastoderma edule*) of the two stocks, naïve (Noia) and affected (Lombos do Ulla), throughout the growing‐out stage in the shellfish beds of Ariño and Sarrido.

Cumulative mortality of cockles from the naïve (Noia) and affected (Lombos do Ulla) stocks in the shellfish beds of Sarrido and Ariño is shown in Figure 2. In Sarrido, the mortality of cockles of the naïve stock increased quickly, reaching almost 90% in the fifth month (April 2021), which impeded further monitoring. In contrast, the cumulative mortality of the affected stock was below 50% 9 months after deployment, when the monitoring finished (August 2021). In the case of Ariño, the cumulative mortality of naïve cockles increased rapidly approaching 90% in the fourth month (March 2021), while in the affected stock, the mortality stayed low and similar to Sarrido until February 2021, but it abruptly increased in the fourth month (March 2021), reaching almost 90% after 5 months (April 2021), which impeded further monitoring.

**FIGURE 3:**
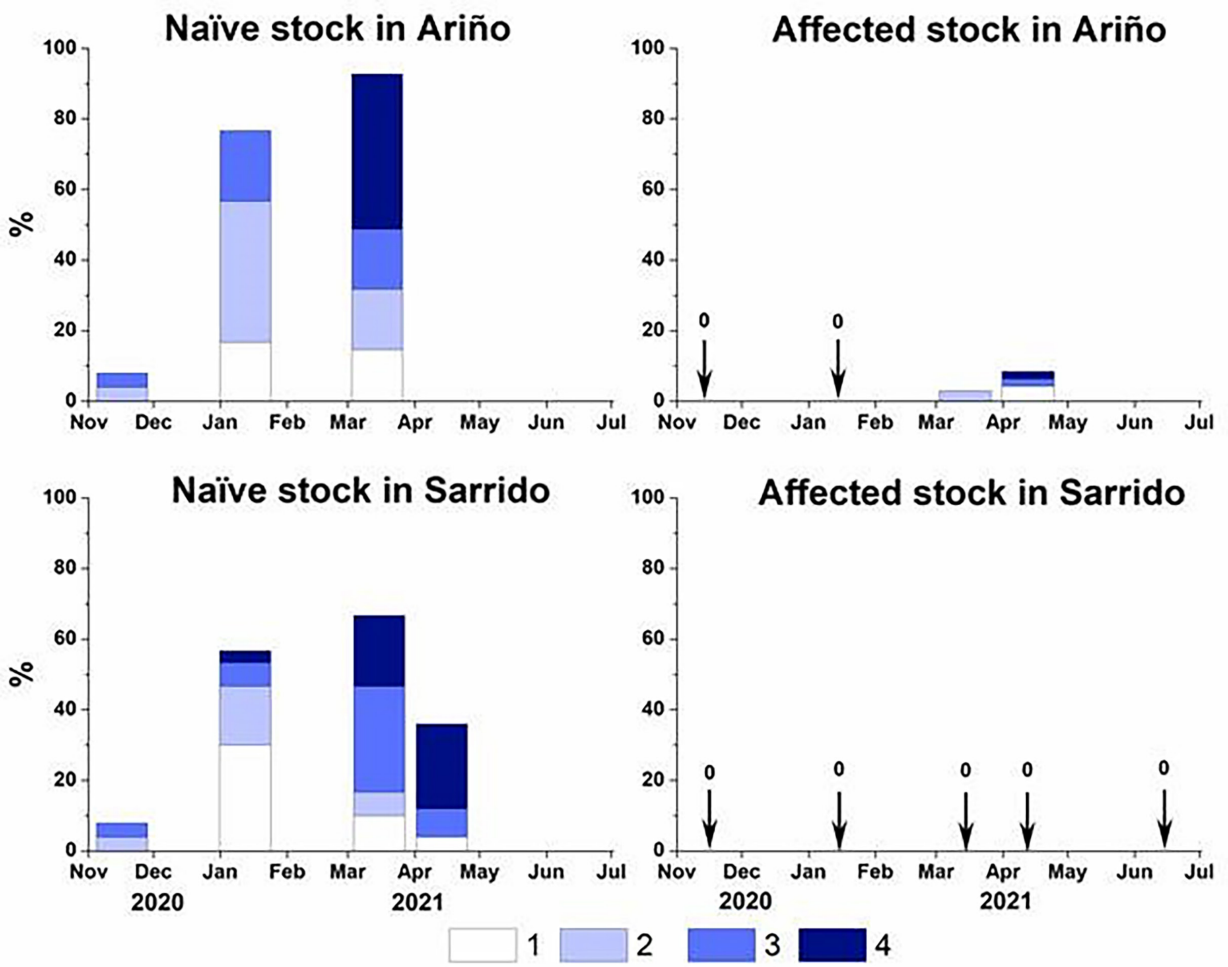
Marteiliosis severity (bars) of cockles (*Cerastoderma edule*) of the two stocks, naïve (Noia) and affected (Lombos do Ulla), through the growing‐out stage in the shellfish beds of Ariño and Sarrido. The height of the bars corresponds to marteiliosis prevalence. The marteiliosis severity stages are distinguished with different colors. Samples in which marteiliosis was not detected (prevalence = 0) are pointed out with arrows.

Regarding marteiliosis prevalence and severity, around 13% of the cockles of the naïve stock were infected when transferred from the raft to the shellfish beds (November 2020), indicating that some became infected during the early grow‐out phase, whereas no infection was detected in the affected stock at that time (Figure 3). In Sarrido, both prevalence and severity of marteiliosis increased in the following months and, in March 2021, more than 70% of the naïve cockles were infected, half of them showing severe (3 and 4) infection stages, which was consistent with the high mortality observed. Remarkably, no case of marteiliosis was detected in the cockles of the affected stock at any time in this shellfish bed (Figure 3). In the Ariño bed, the prevalence and severity of marteiliosis increased even faster than in the outer shellfish bed and, in March 2021, more than 90% of the cockles from the naïve stock were infected. Around half of them showed severe (stage 4) infection (Figure 3), which was consistent with the very high mortality detected. Conversely, marteiliosis was detected in the affected stock only in March 2021 (3.0% prevalence) and April 2021 (8.5% prevalence). Those values of marteiliosis prevalence were low and, consequently, this disease cannot be responsible for the high mortality observed in the affected stock in Ariño in those months. Other cockle diseases with potential lethality reported in the Galician rias, such as disseminated neoplasia (Díaz et al., 2016), infection with haplosporidan parasites (Azevedo et al., 2003; Carballal et al., 2020; Ramilo et al., 2018), and with trematode sporocysts (Carballal et al., 2001) showed low prevalence, without significant differences with those from Sarrido bed. Interestingly, the cockles of the affected stock collected from Ariño in March and April 2021 presented histological stress signs, such as heavy hemolytic infiltration of the connective tissue in most organs as well as gonadal regression, which were not observed either in the previous months in the same bed or in the cockles of the affected stock deployed in Sarrido. The causes of those stress signs were not identified but the location of the Vilanova de Arousa harbor and some seafood processing factories near the shellfish bed of Ariño suggested the hypothesis that toxic spills might have contributed to the abnormal abrupt mortality observed.

### 3.2 Genotyping and validation of the multiplex SNP tool

MassARRAY genotyping of the selected SNPs, previously associated with marteiliosis resilience (Pampín et al., 2023), confirmed the allelic variants identified from RAD‐seq data. Nevertheless, among the 45 SNPs included in two MassARRAY multiplexes (Table S1), four were monomorphic (Table S2), when these SNPs had shown *H*_E_ values between 0.117 and 0.440 in a previous study in the same location (Lombos do Ulla; Pampín et al., 2023). Further, other five SNPs showed missing genotyping values >20% (between 23% and 60%; Table S2). Technical issues related to primer design on polymorphic genomic regions or to genome assembly could explain these outcomes and technical refinement should be performed for their application in future studies. Anyway, all the information was retained for further analyses and these SNPs were only excluded for multiple correspondence analysis, where missing data pose a serious issue.

### 3.3 Genetic diversity in the naïve and affected stocks

**FIGURE 4:**
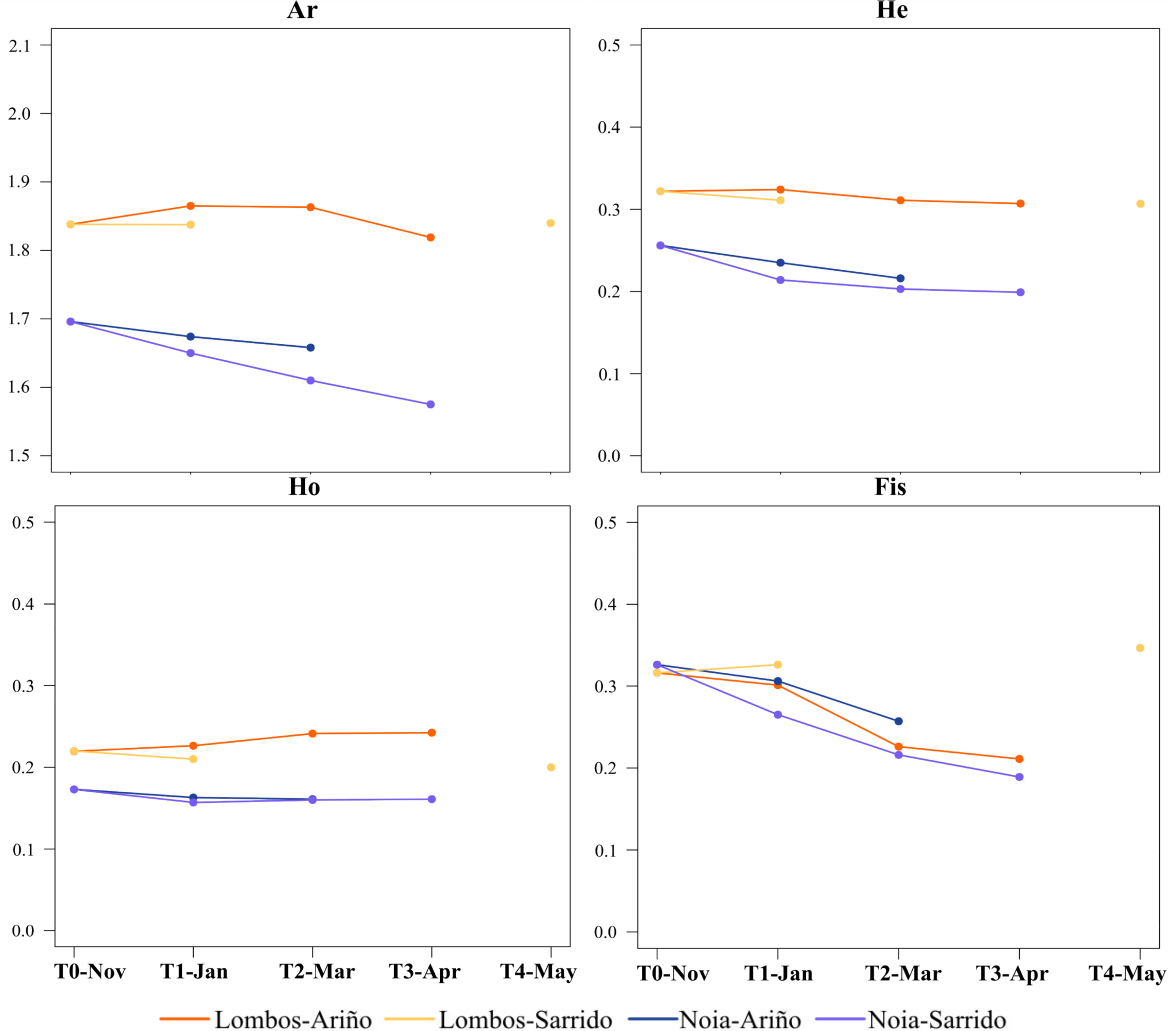
Genetic diversity estimators in samples from the common garden experiment carried out in two stocks of *Cerastoderma edule*, Noia (naïve) and Lombos do Ulla (affected), settled in two affected shellfish beds of Ría de Arousa (Sarrido and Ariño) through the growing‐out stage, from November 2020 (T0‐Nov) until May 2021 (T4‐May) (see Table 1). Ar, allelic richness; *H*_E_, expected heterozygosity; *H*_O_, observed heterozygosity; *F*_IS_, intrapopulation fixation index.

Expected heterozygosity (*H*_E_) estimated with the 45 SNPs for the Lombos do Ulla stock was moderately high for biallelic markers (*H*_E_ = 0.322). All estimators consistently showed higher genetic diversity figures in the samples from the affected stock (Lombos do Ulla) than those from the naïve stock (Noia): Ar (1.838 vs. 1.696), *H*_E_ (0.321 vs. 0.256), and *H*_O_ (0.220 vs. 0.173) (Wilcoxon rank paired tests *p* < 0.001 in all cases) (Tables S3 and S4; Figure 4). However, in a previous study, cockle samples from the same shellfish beds of Ría de Arousa and Ría de Muros‐Noia did not show genetic diversity differences using a 9250 SNP 2b‐RADseq panel (Vera et al., 2023). Since these 45 SNPs were specifically selected for their association with marteiliosis resilience in the affected stock (Pampín et al., 2023), the most likely explanation is that they are located in genomic regions differentiated from the neutral background. In fact, several SNPs showed very high (*H*_E_ = 0.5: CE5, CE30, and CE31) to moderate (*H*_E_ = 0.3: CE1 and CE14) genetic diversity in the affected stock, while they were nearly fixed in the naïve stock (*H*_E_ = 0) (Figure S3).

We also observed significant heterozygote deficit in samples from both stocks (*F*_IS_ = 0.316 and 0.326 in affected and naïve stocks, respectively) (Tables S3 and S4; Figure 4). Positive *F*_IS_ values have been commonly reported in mollusks (Pino‐Querido et al., 2015) and specifically in common cockle (Vera et al., 2022), and have been mostly attributed to the presence of null alleles related to the high nucleotide polymorphisms reported in this group (Guo et al., 2023; Zhan et al., 2009). However, *F*_IS_ values in our study were significantly higher than those previously reported in shellfish beds from Galicia (average *F*_IS_ = 0.104; Vera et al., 2023) and could be either related to the mixing of different cohorts originated from small number of contributors when obtaining progenies at hatchery (Wahlund effect) or to the particular high heterozygote deficit detected at specific loci, such as CE2, CE11, CE25, CE50, CE51, CE52, and CE53 (Figure S4).

Interestingly, *H*_E_ consistently decreased along the growing‐out stage in the naïve stock in both shellfish beds from the initial sampling coming from the raft (pairwise Wilcoxon rank paired test Sarrido: *p*_N vs. NS1_ = 0.0105, *p*_N vs. NS2_ = 0.0056, *p*_N vs. NS3_ = 0.0071; Ariño: *p*_N vs. NA1_ = 0.0905, *p*_N vs. NA2_ = 0.0144; see Table 1 for codes), suggesting that it could be related to directional selection on the SNPs analyzed driven by marteiliosis (Table S4; Figure 4). This trend was not observed in the affected stock, in which marteiliosis prevalence was null (in Sarrido) or very low (in Ariño). Specific loci such as C8, C11, CE13, and CE18 reflected very well this general trend throughout the temporal series of the naïve stock (Figure S3, Table S3). A similar decreasing trend was also observed for *F*_IS_, indicating that the proportion of observed heterozygotes was maintained throughout the growing‐out stage in the naïve stock. This suggests a dominance or overdominance component for marteiliosis resilience on the selected SNPs (Table S4; Figure 4), reflected at specific loci (CE3, CE18, C35, CE39, and CE44; Figure S4). However, the statistical support for *F*_IS_ was only suggestive and exclusively detected in the Sarrido shellfish bed (pairwise Wilcoxon rank paired tests Sarrido: *p*_N vs. NS1_ = 0.5291, *p*_N vs. NS2_ = 0.0739, *p*_N vs. NS3_ = 0.0144; Ariño: *p*_N vs. NA1_ = 0.2241, *p*_N vs. NA2_ = 0.1579).

### 3.4 Genetic differentiation between stocks and along the growing‐out stage

The global relative component of genetic differentiation (*F*_ST_) for the 45 SNPs across all studied samples was highly significant (null hypothesis *F*_ST_ = 0; *F*_ST_ = 0.073, *p* < 0.001). Global *F*_ST_ per locus ranged from 0 to 0.265 and was significant for most SNPs (monomorphic loci excluded; 28/41: 68.3% after Bonferroni correction [*p* < 0.0013]). The partition of this global *F*_ST_ value showed much higher divergence between (8.28%) than within (1.51%) stocks (Table S5a). When partition was assessed within stocks, much higher differentiation was detected among samples within the naïve stock than within the affected stock (3.03% vs. 0.71%) (Table S5b,c). A very similar pattern was observed when analyzing pairwise *F*_ST_ values (Table S6).

**FIGURE 5:**
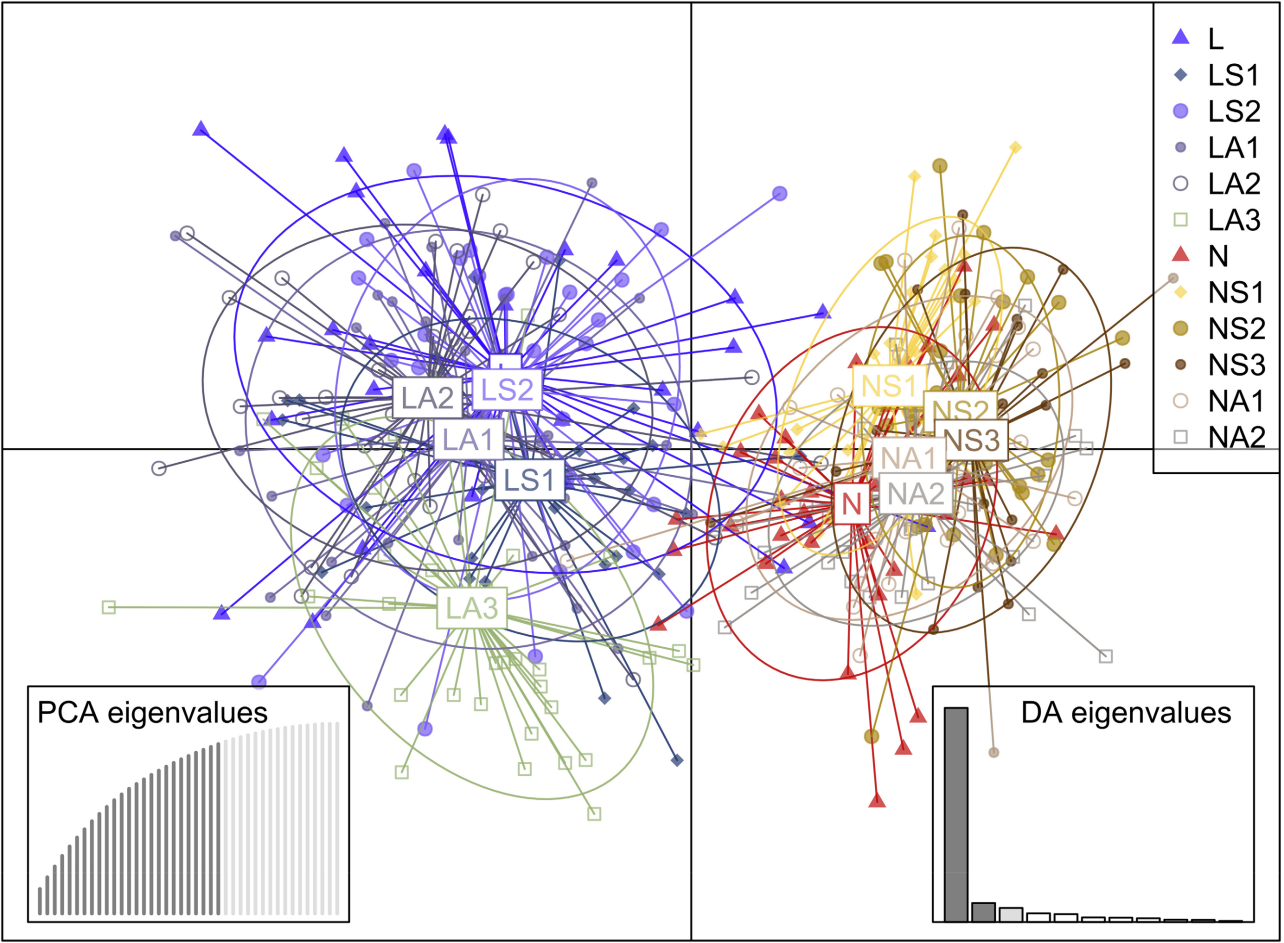
Discriminant analysis of principal components (DAPC) of *Cerastoderma edule* samples from a common garden experiment carried out in Ría de Arousa using two stocks with different marteiliosis status, naïve (Noia) and affected (Lombos do Ulla), using 45 candidate SNPs previously associated with marteiliosis resilience (Pampín et al., 2023). The weight of the two retained discriminant analyses (DA) and 25 PCA eigenvalues representing 89% of the variance are shown on the bottom boxes. Sample codes are shown in Table 1.

The structure analysis was completed with Bayesian (STRUCTURE) and multivariant (DAPC) statistical methods without a priori information. STRUCTURE rendered the best value of *K* = 2, according to the Δ*K* method of Evanno et al. (2005), separating the affected (Lombos: blue cluster) and naïve (Noia: orange cluster) stocks (Figure S5). DAPC showed a similar segregation of the affected (Lombos do Ulla) and naïve (Noia) stocks (Figure 5).

### 3.5 Relationship among variables: Stock, infection, and SNPs

**FIGURE 6:**
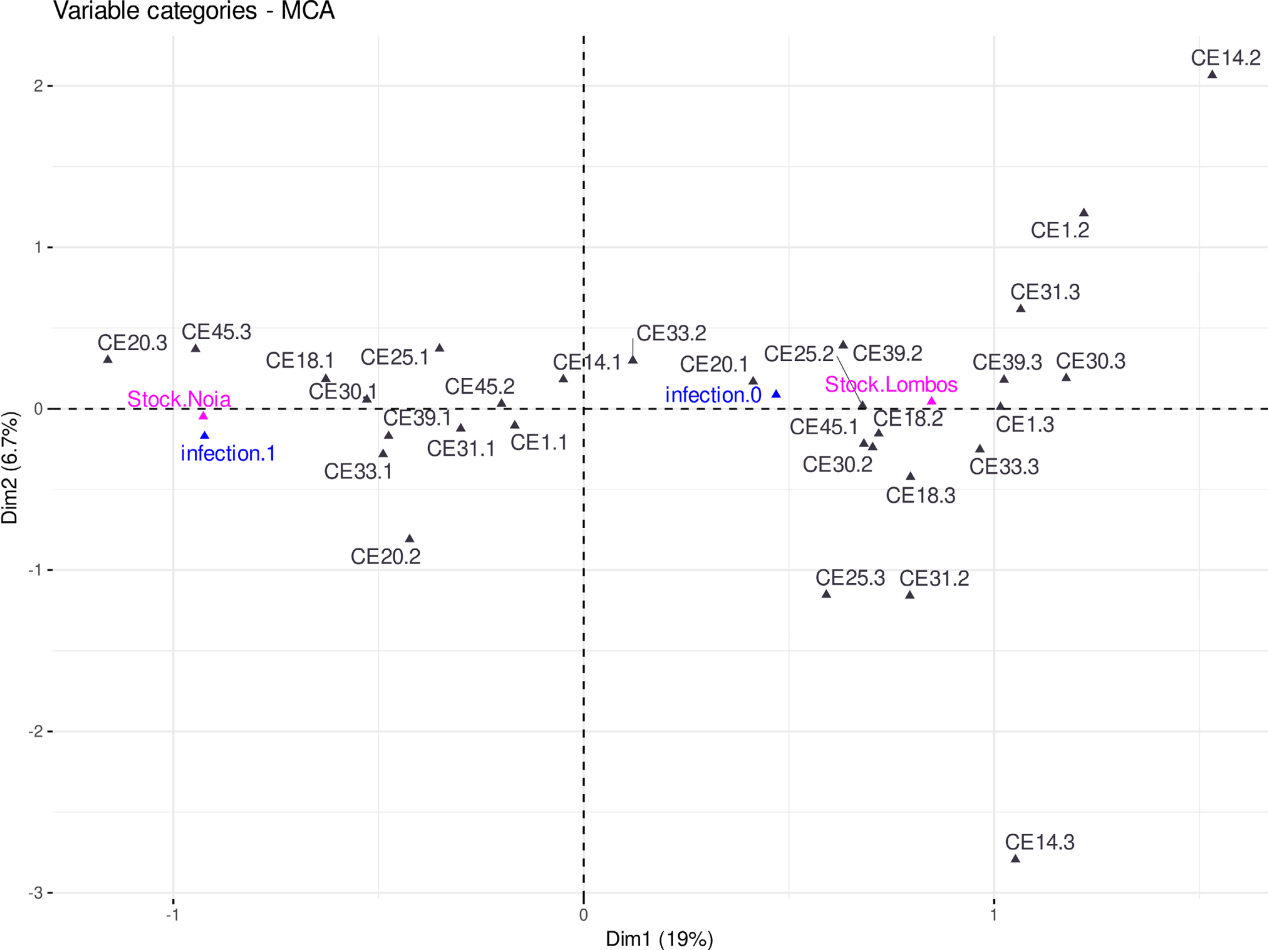
Multiple correspondence analysis (MCA) in the common garden experiment of *Cerastoderma edule* showing the relationship among the different variables studied (stock: Lombos do Ulla and Noia; infection status: non‐infected [0] and infected [1); the 10 most discriminant SNPs [CE1, CE14, CE18, CE20, CE25, CE30, CE31, CE33, CE39 and CE45]; genotypes: homozygotes [1 and 3] and heterozygote [2]).

We explored the relationship among the variables studied in the common garden experiment, to say, the SNP markers, the stocks (naïve and affected), and the infection status (non‐infected [0] and infected [1]) using multiple correspondence analyses. The percentage of explained variance of the two first dimensions decayed from the five most discriminant SNPs (*F*_ST_ between 0.138 and 0.265; first dimension 26.4%, second dimension 9.6%; Figure 6) down to the total 38 SNPs used in the analysis (*F*_ST_ between 0 and 0.265; first dimension 7.9, second dimension 4.1%), showing the progressive loss of statistical power as the number of less discriminant markers increased. We inspected the plot obtained with the 25 SNPs (missing data <0.20) showing significant genetic differentiation after Bonferroni correction between the naïve and affected stocks (*F*_ST_ between 0.030 and 0.265; *p* < 0.0013; Figure S6a) and a clear association of the affected stock (Lombos do Ulla) with no infection, and the naïve stock (Noia) with the infection status, was shown. Also, though more difficult to visualize because of the amount of genotyping data, heterozygotes plotted mostly around the Lombos/infection.0 area of the plot (19 loci), only two heterozygotes being close to the Noia/infection.1 area and a few ones located in‐between (five loci), suggesting dominance effects associated with resilience to the parasite for most of the markers (Figure 6). This fact was easier to visualize when the 10 most discriminating SNPs between naïve and affected stocks were evaluated (*F*_ST_ between 0.165 and 0.265; Figure 6). Genotypes of the 10 SNPs were distributed along the first dimension and most heterozygotes plotted around the non‐infected status (0), excluding CE20 and CE45.

We also intended to unveil signals of selection in the short‐term using genotyping information along the growing‐out stage in the naïve (Noia) stock, the only stock heavily affected by marteiliosis. To increase the statistical power, we aggregated individuals of the two shellfish beds according to their infection status, since they can be considered replicates of the same outbreak occurring in Ría de Arousa. Six markers, CE2, CE8, CE18, CE20, CE51, and CE53, displayed significant differentiation after Bonferroni correction among samples sorted by infection degree (*F*_ST_ from 0.035 to 0.120; *p* < 0.0016), all of them, being also among the 25 significant detected in the long‐term comparison (naïve vs. affected stocks). The first two dimensions explained 16.0% and 11.6% of the variance, respectively (Figure S6b), and the infection and non‐infection status appeared separated in the plot. Association of genotypes with the infection status was not so obvious as in the long‐term selection multiple correspondence analysis, and the effect of dominance was more difficult to observe in this scenario.

### 3.6 Signals of selection: Candidate genes

A subset of SNPs, such as CE3, CE6, CE22, CE23, CE35, CE36, CE44, CE50, and CE52, displayed high genetic diversity (*H*_E_ ~ 0.5), but not significant genetic differentiation among samples or stocks (*F*_ST_ = 0) (Figure S3, Table S3). Despite being located within important genes related to immune response (cathepsin), lipid trafficking and metabolism (cholesterol 25‐hydroxylase‐like, oxysterol‐binding protein), and neural transmission (HMG box‐containing protein C19G7.04‐like, sodium‐ and chloride‐dependent glycine transporter 1‐like), they were not apparently associated with selection against marteiliosis both in the long‐term (naïve vs. affected) or in the short‐term (along growing‐out stage in the naïve stock). However, some of the 25 SNPs confidently differentiating naïve and affected stocks were located within relevant immune‐related genes, such as 26S proteasome non‐ATPase regulatory subunit 9‐like, metalloendopeptidase, tumor necrosis factor ligand superfamily member 10, E3 ubiquitin–protein ligase HERC4, and glutathione S‐transferase sigma class, while others were associated with neural function and development, such as synaptojanin‐1‐like. Although some of the 25 SNPs clustered at specific chromosomes (three SNPs at chromosomes 3, 6, 10, and 14), they were several megabases away, so not being part of particular genomic regions under selective pressure, especially considering the small average linkage disequilibrium reported in the Atlantic area for common cockle (Vera et al., 2022).

## 4 DISCUSSION

Mollusk production is very diversified and represents 1/3 of the world aquaculture (FAO, 2021). It includes species cultured under intensive or extensive programs, depending on seed production at hatchery or from natural collection (Moor et al., 2022). Diseases represent an important concern for mollusk producers and their control is complex since production takes place in an open environment. Emergent pathogens, which have increased with the effects of climate change, have accentuated this problem. Pathologies are among the most important factors driving selection in wild and domestic populations and thus shaping their genomes (Consuegra et al., 2015; Karlsson et al., 2014; Potts et al., 2021). Identification of genetic variation underlying resilience to pathogens is essential to understand evolutionary processes and managing natural resources, but also to improve animal production (Bishop & Woolliams, 2014; Gjedrem & Rye, 2018; Guo, 2021).

Marteiliosis has devastated the common cockle production in Galicia, the main mollusk production region of Spain. Previous studies suggested that marteiliosis resilience increased across generations in the cockle beds of the inner area of Ría de Arousa (Iglesias et al., 2023, in press). The results of our common garden experiment showed very high prevalence of marteiliosis in the naïve stock in both shellfish beds, whereas the prevalence in the affected stock was null (Sarrido) or very low (Ariño). Our experimental design ensured the same environmental conditions for the cockles of the two stocks, particularly the same exposure rates to *M. cochillia*, thus demonstrating a much higher resilience of the affected stock to marteiliosis. Furthermore, the affected stock became nearly resilient to the parasite in a short period of 8 years, encompassing eight generations maximum, assuming cockle's sex maturity during the first year of life (Maia et al., 2021). This rapid adaptive process is consistent with the severe fitness effects of marteiliosis, which affects both adult and juvenile stages (Iglesias et al., 2023; Villalba et al., 2014). Other examples of fast resilience acquisition against pathogens in the wild have been reported, including parasite resistance to whirling disease in wild rainbow trout from Harrison Lake (Montana, USA) in less than 7 years (1995–2002) (Miller & Vincent, 2008) and the resistance to wasting disease (putatively caused by a virus) in sea star *Pisaster ochraceus*, which was almost decimated in the American Pacific coast in 2013 (Schiebelhut et al., 2018). Disease resilience can be due to resistance to be infected and/or to tolerance once the pathogen has infected individuals; while resistance reduces pathogen load, tolerance improves survival by repairing the damage caused by the parasite (Agnew et al., 2020; Bailey et al., 2019; Carvalho et al., 2012; Horns & Hood, 2012). Both mechanisms can increase the fitness of the infected organisms (Roy & Kirchner, 2000) and can evolve fast in the wild under strong pathogen pressure. In our study, since *M*. *cochillia* was hardly detected in the samples of the affected stock analyzed histologically, we hypothesize that the mechanism for increasing cockle resilience to marteiliosis is more likely related to resistance rather than tolerance.

Understanding the genetic basis of disease resilience can be tackled through different genomic approaches, such as the identification of outlier loci associated with resilience against the neutral genomic background through a population genomics approach or the identification of differentially expressed genes (DEG) and SNP markers associated with the infection degree, which can eventually lead to the identification of the causal mutations (Potts et al., 2021; Vera et al., 2019). In a previous study, Pampín et al. (2023) identified a set of SNP markers associated with resilience to *M*. *cochillia* following a combination of population genomics (110 SNPs) and transcriptomic (123 SNPs) approaches in Ría de Arousa, the same scenario where our common garden study was performed. Starting from this information, we designed a cost‐effective MassARRAY genotyping tool including the most interesting SNPs in two multiplexes to ascertain their association with marteiliosis resilience. Most SNPs, excluding four monomorphic, were consistent with the previous 2b‐RADseq genotyping (91%), although five of them showed >20% of missing data and should be technically improved in future studies. The common garden design enabled us to validate the association of the selected SNPs with marteiliosis resilience in a new sampling scenario and to ascertain their role both in the long term, by comparing the genetic differentiation between naïve and affected stocks, as well as in the short term, by analyzing the growing‐out stage in the naïve stock in two shellfish beds. It is important to point out that no genetic differentiation was detected between samples from Ría de Muros‐Noia and Ría de Arousa, where shellfish beds of naïve and affected stocks are, respectively, located using a genomic screening with ~9000 SNPs (*F*_ST_ not ≠ 0; Vera et al., 2023). This genetic homogenization has been attributed to the high dispersal capacity of cockles during planktonic free‐living stages and to its large population size (Vera et al., 2022).

Unlike the genomic screening by Vera et al. (2023), we observed a highly significant genetic differentiation with the 45 selected SNPs between Noia and Lombos do Ulla stocks (*F*_ST_ = 0.082, *p* = 0) and the genetic structure analysis without a priori information (STRUCTURE, DAPC) identified two main clusters associated with the naïve and the affected stocks, respectively. Strong selective pressure, such as that caused by *M*. *cochillia* in Ría de Arousa, could leave signatures at specific genomic regions against the homogenizing trend of migration, reflected by significant genetic differentiation between samples, as reported in other species (Nielsen et al., 2009; Vilas et al., 2015). Accordingly, despite migration of cockle larvae from the naïve Northern Rías (e.g., Ría de Muros‐Noia) to Ría de Arousa could decrease the frequency of resilient genotypes, the strong selective pressure would maintain differentiation at specific genomic regions. These results confirm the previous observations by Pampín et al. (2023) in the 2018/2019 marteiliosis outbreak in Ría de Arousa and endorse the SNP strategy performed to identify genetic markers and candidate genes associated with marteiliosis resilience. However, while most selected SNPs (68.3%) showed significant differentiation between both naïve and affected stocks after Bonferroni correction (*F*_ST_ range 0.030–0.265; *p* < 0.0013), other SNPs, mostly coming from the transcriptomic approach, showed no differentiation and could be false positives. The sample size used by Pampín et al. (2023) to estimate genetic differentiation on SNPs within DEGs between samples with increasing infection status was not large enough, so this is not an unexpected outcome. Our data also suggest a dominant component associated with the resilience to marteiliosis in the most discriminant SNPs, which would delay the removal of the susceptible allele, especially considering the short time since the appearance of marteiliosis in Ría de Arousa. Selection against a recessive allele depends on the coefficient of selection, allele frequency, and the number of generations elapsed. Even in the most extreme scenario, to say assuming a coefficient of selection (s) = 1, the number of generations since the beginning of marteiliosis in 2012 would be low, especially considering overlapping of generations due to the lifespan of cockles (up to 10 years). Among the most discriminating SNPs, several were located within relevant mollusk immune genes such as E3 ubiquitin–protein ligase HERC4 (de la Ballina et al., 2021; Lin et al., 2022; Vaibhav et al., 2018; Zhang et al., 2016), tumor necrosis factor ligand superfamily member 10 (Liu et al., 2021), metalloendopeptidase (Guo & Ford, 2016), 26S proteasome non‐ATPase regulatory subunit 9‐like (de Lorgeril et al., 2011), and glutathione S‐transferase sigma class (Ren et al., 2009; Umasuthan et al., 2012; Vodiasova et al., 2021; Wang et al., 2018; Yang et al., 2012); and to nervous system development and function, such as synaptojanin‐1‐like (Choudhry et al., 2021). We also explored the putative effects of marteiliosis on the genetic constitution of common cockle in the short term by analyzing grow‐out stage of the naïve stock in two shellfish beds. Although not so intense as in the long‐term evaluation, as expected, we detected significant decrease of genetic diversity and increase of genetic differentiation among samples with different infection degrees across the growing‐out phase. Six SNPs, also identified in the long‐term selection scenario, showed significant differentiation associated with the infection degree (*F*_ST_ range: 0.035–0.120; *p* < 0.0016). A similar strategy for disentangling the role of natural selection over multiple sites across time has been successfully applied to ascertain the resistance to the wasting disease in *Pisaster ochraceus* using 100 top outliers discriminating pre and post outbreak adult survival (Schiebelhut et al., 2018).

## 5 CONCLUSIONS AND PERSPECTIVES

Our results demonstrated the rapid marteiliosis resilience acquired by common cockle through natural selection in a short period of time. We also demonstrated the genetic divergence from naïve and affected stocks for the selected SNPs against the non‐differentiated genomic background previously reported. These SNPs are involved in different biological functions, especially related to immune system, and they do not colocalize at any specific genomic region but seem to be scattered across different chromosomes, which suggests an underlying polygenic nature. Although this makes more complex obtaining resilient strains at hatchery, one MassARRAY multiplex combining the most discriminant SNPs could be applied and tested at hatchery for this purpose. Specific genes such as those related to immunity differentiating infected and non‐infected stocks or individuals would deserve in‐depth functional evaluation related to marteiliosis resilience. It should be emphasized that our study was focused on genetic markers detected by their association with divergent selection (naïve vs. affected stocks; non‐infected vs. infected individuals), but we cannot discard other models of selection operating in our scenario, such as overdominance, which has demonstrated to be involved in tolerance or resistance to stress or pathologies in other studies (Di et al., 2015; Gallaga‐Maldonado et al., 2020; Maynard et al., 2016). Also, the putative role of epigenetic changes and their transmission across generations (“priming”; Eirin‐Lopez & Putnam, 2019; Fallet et al., 2022; Johnson et al., 2020; Liu et al., 2022; Potts et al., 2021) should be evaluated to understand the quick resilience acquisition against marteiliosis observed in common cockle. In this regard, we have compiled a valuable biological material, both from different wild stocks as well as from families of both stocks obtained at hatchery, to investigate the putative epigenomic “priming” underlying marteiliosis resilience. Currently, a refined SNP panel of the most informative candidates is being applied to assess the results of selection at hatchery in comparison with selection operating in the wild, an essential information for taking decisions on cockle bed management in Galicia.

## CONFLICT OF INTEREST STATEMENT

Authors have no conflicts of interest to declare.

## PUBLICATION

These results are not under consideration to be published in any other journal. All authors have read and approved the manuscript which we submit on their behalf.

## Supporting information


Figure S1.

Figure S2.

Figure S3.

Figure S4.

Figure S5.

Figure S6.

Table S1.

Table S2.

Table S3.

Table S4.

Table S5.

Table S6.

## Data Availability

Genotyping information has been included in Table S2.
