# Supplementary figures and images for: A common garden experiment supports a genetic component underlying the increased resilience of common cockle (Cerastoderma edule) to the parasite Marteilia cochillia
Source: Evol Appl. 2023 Oct 17;16(11):1789–804. doi: 10.1111/eva.13601 (PMC10681494; doi:10.1111/eva.13601)

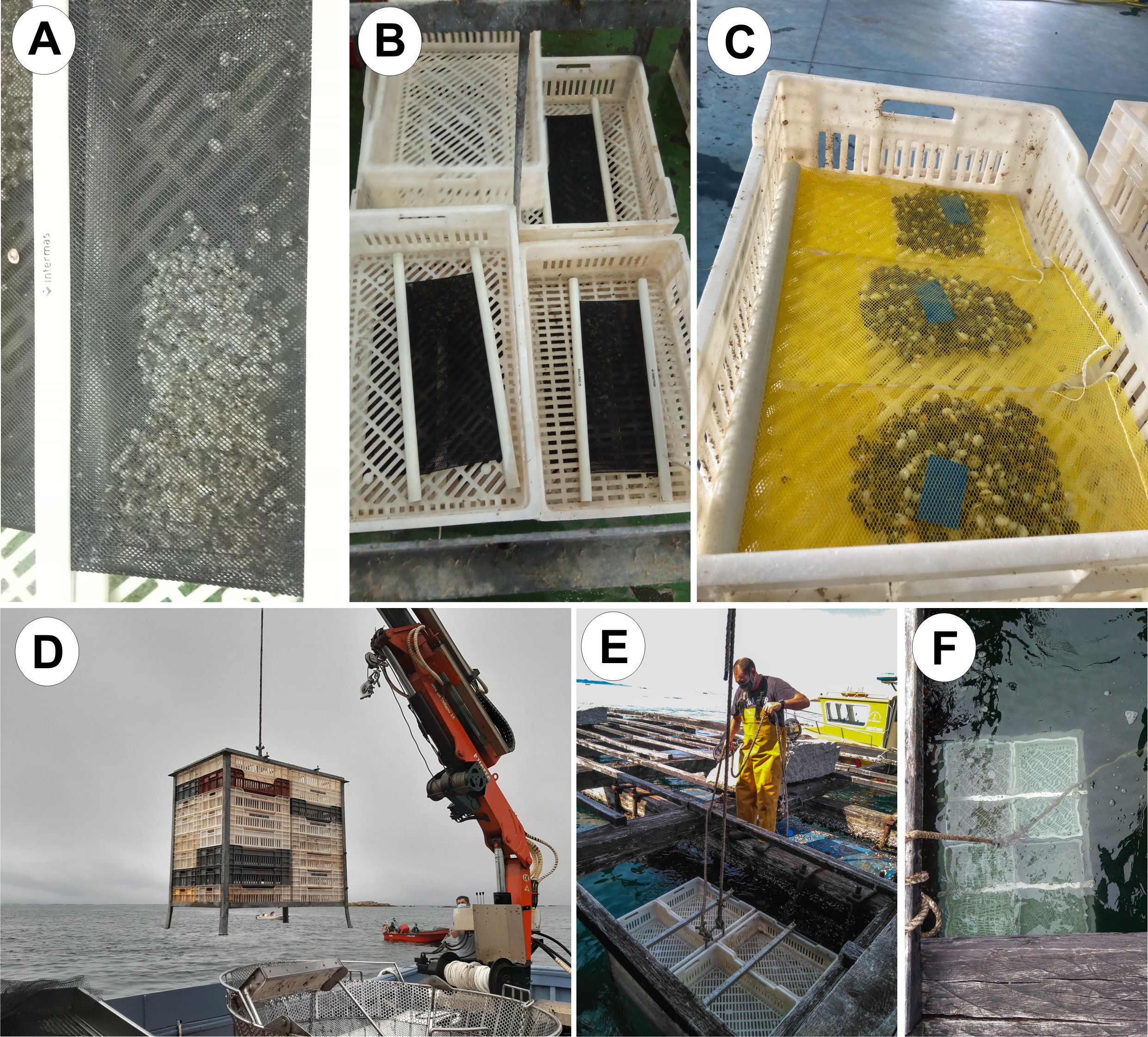

Supplement: Supplementary file 1 — Figure S1. [file EVA-16-1789-s004.jpg]

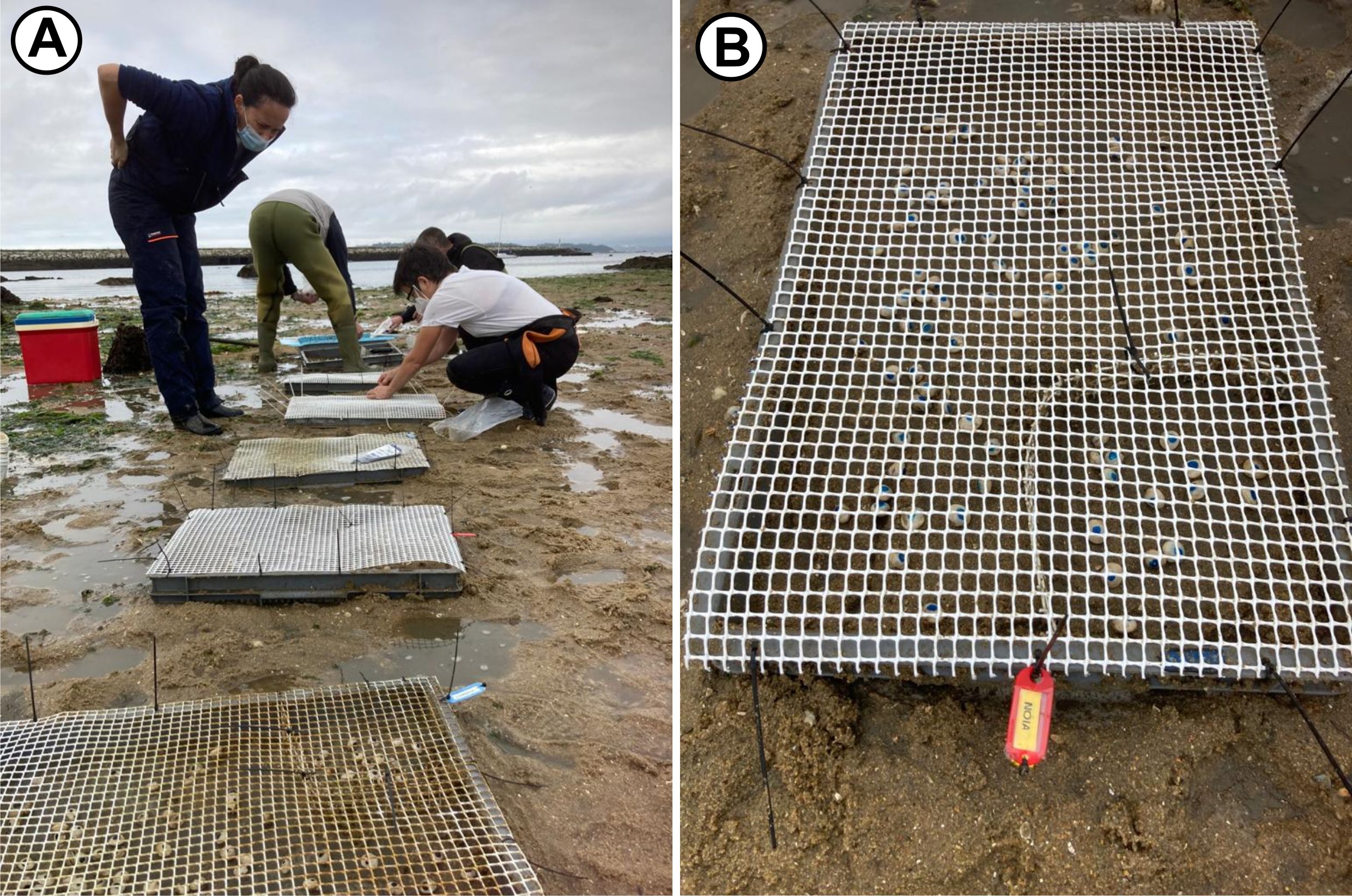

Supplement: Supplementary file 2 — Figure S2. [file EVA-16-1789-s005.jpg]

## Slide 1
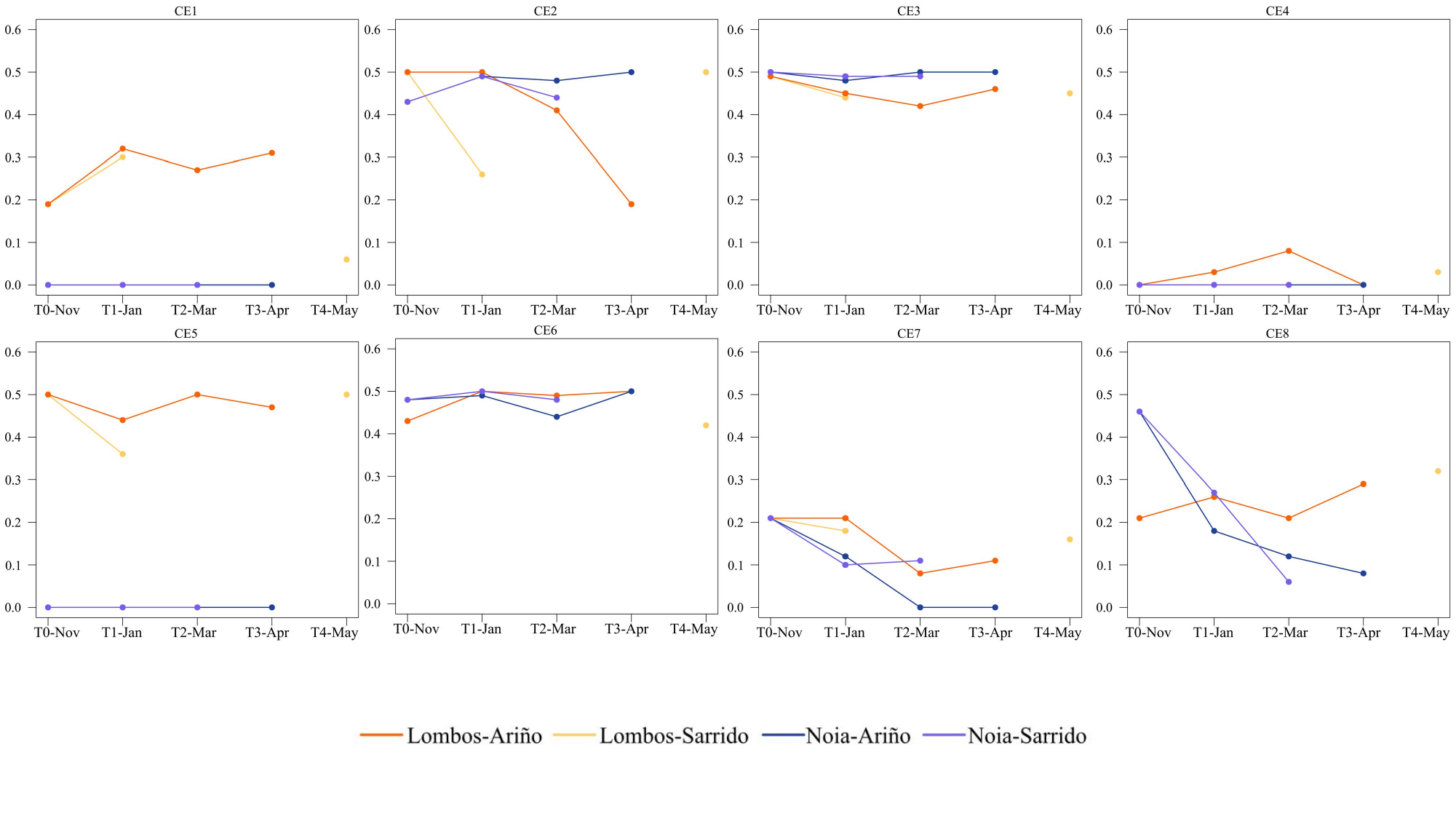

## Slide 2
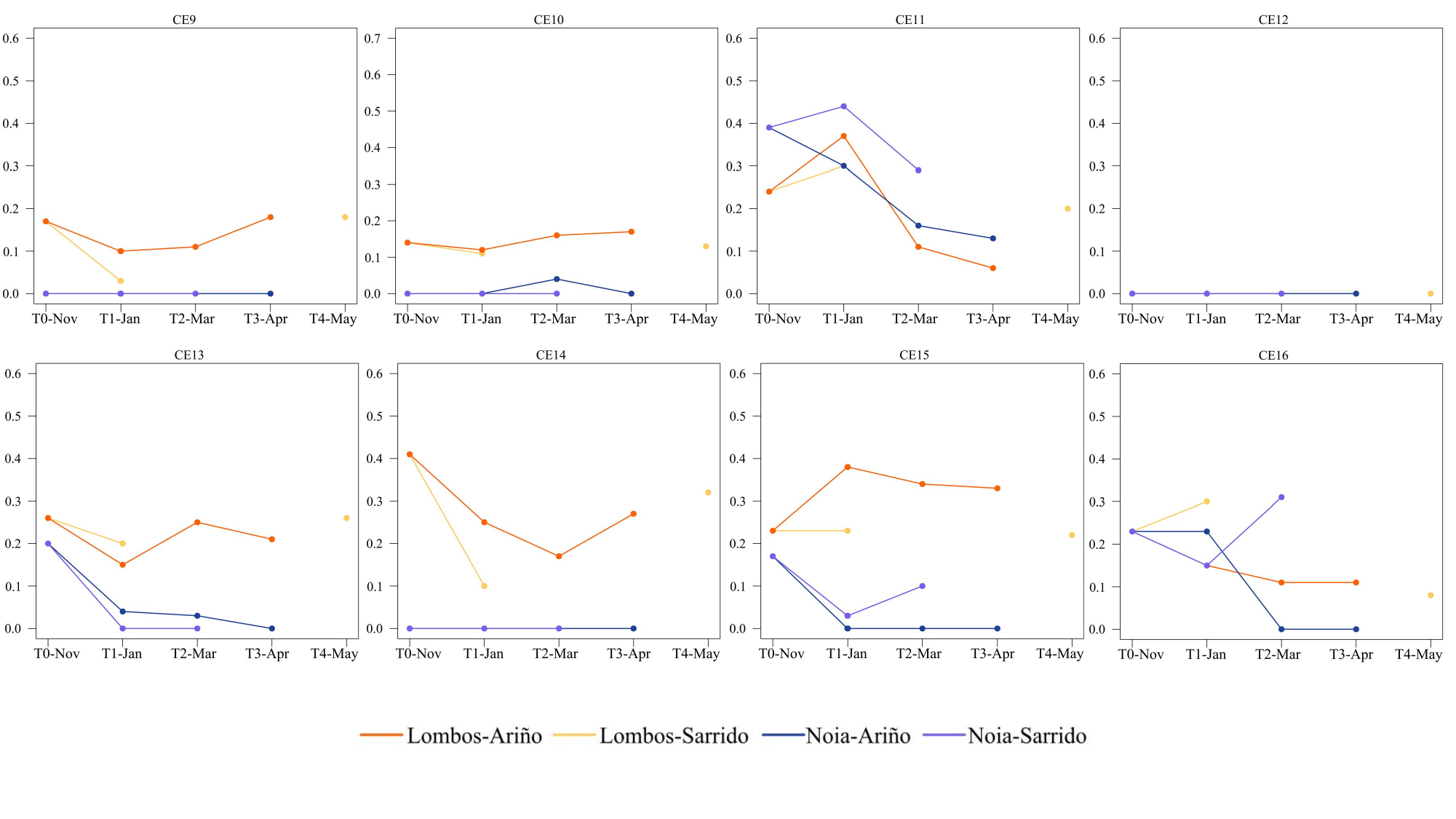

## Slide 3
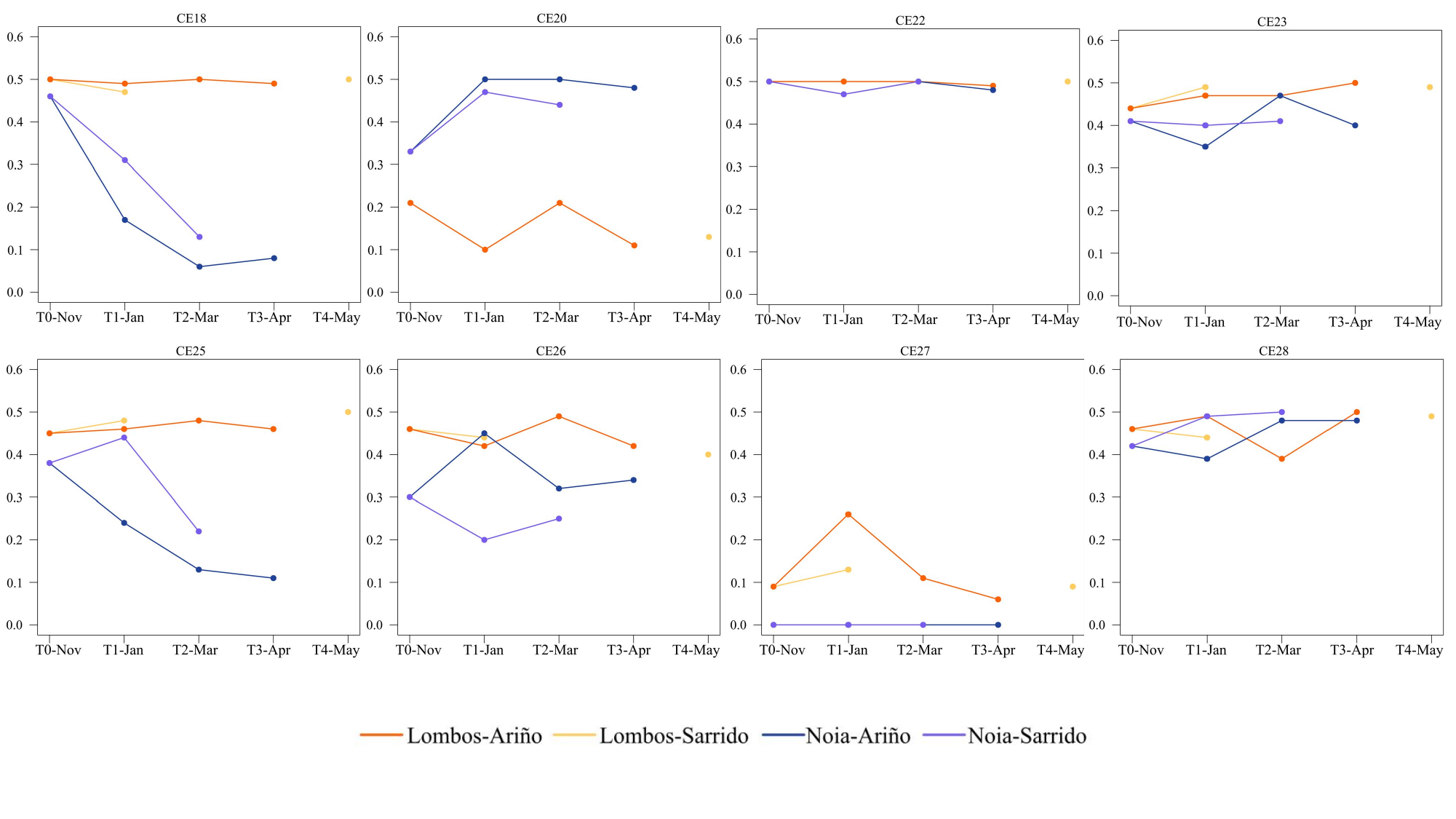

## Slide 4
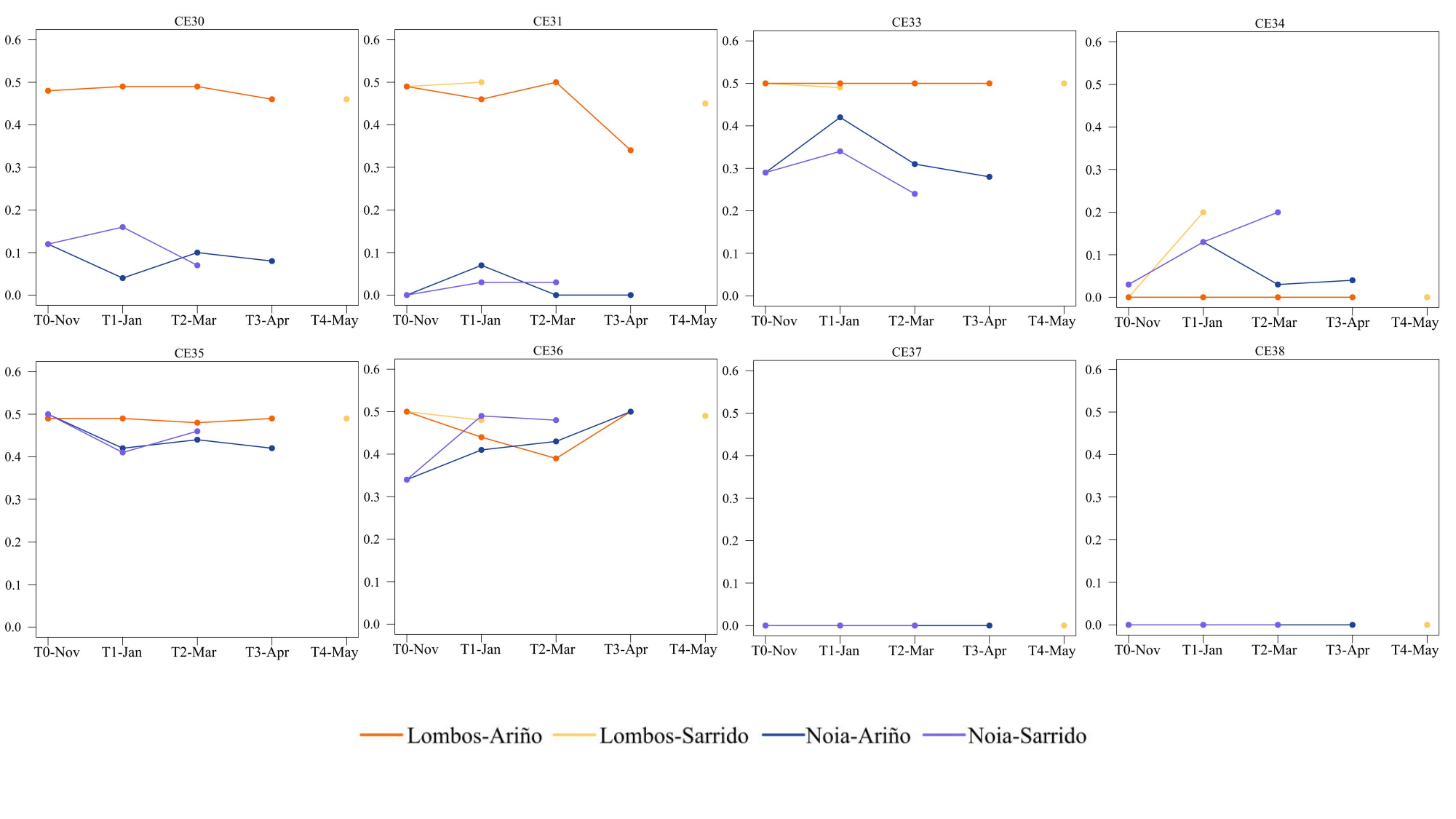

## Slide 5
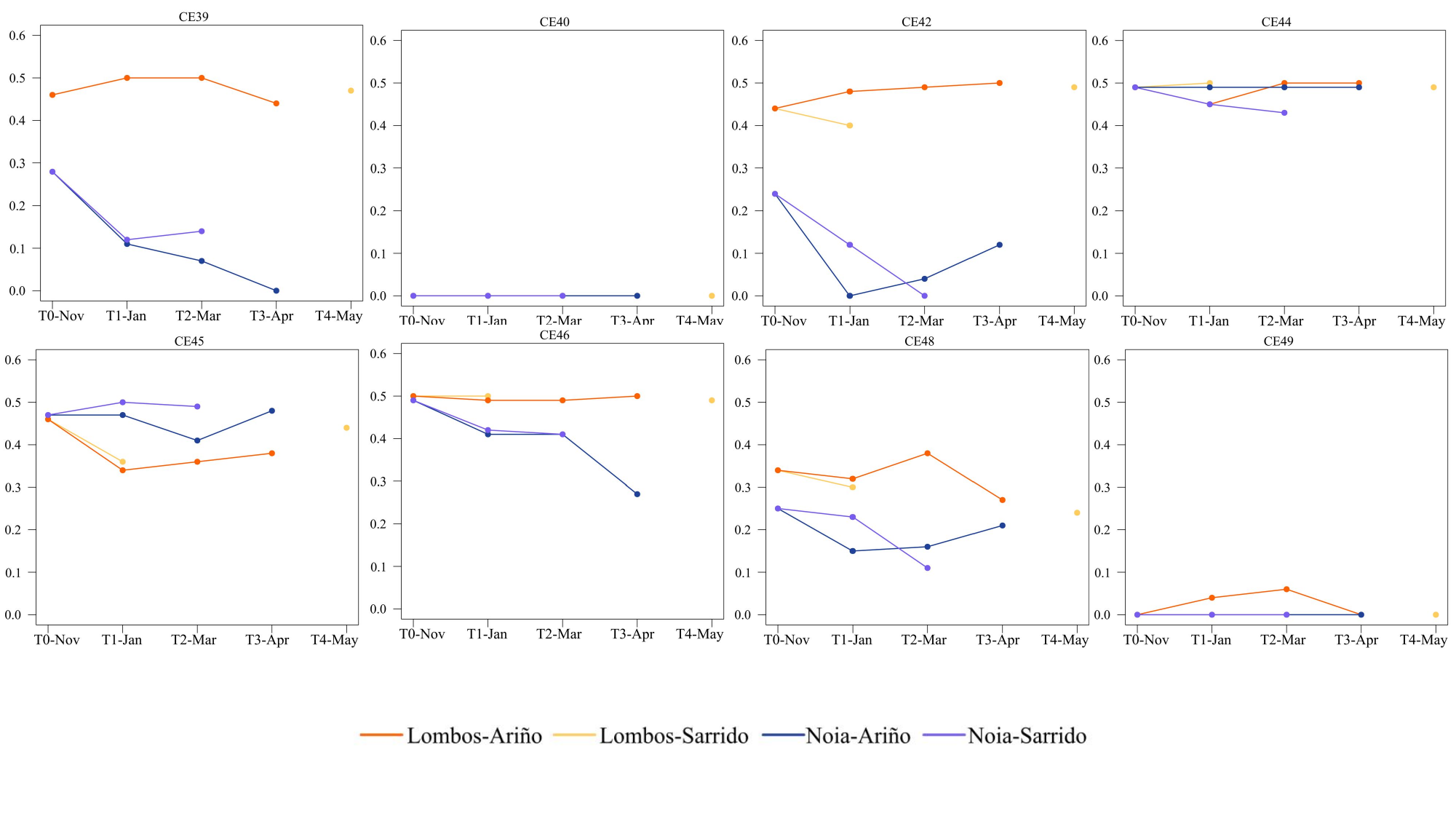

## Slide 6
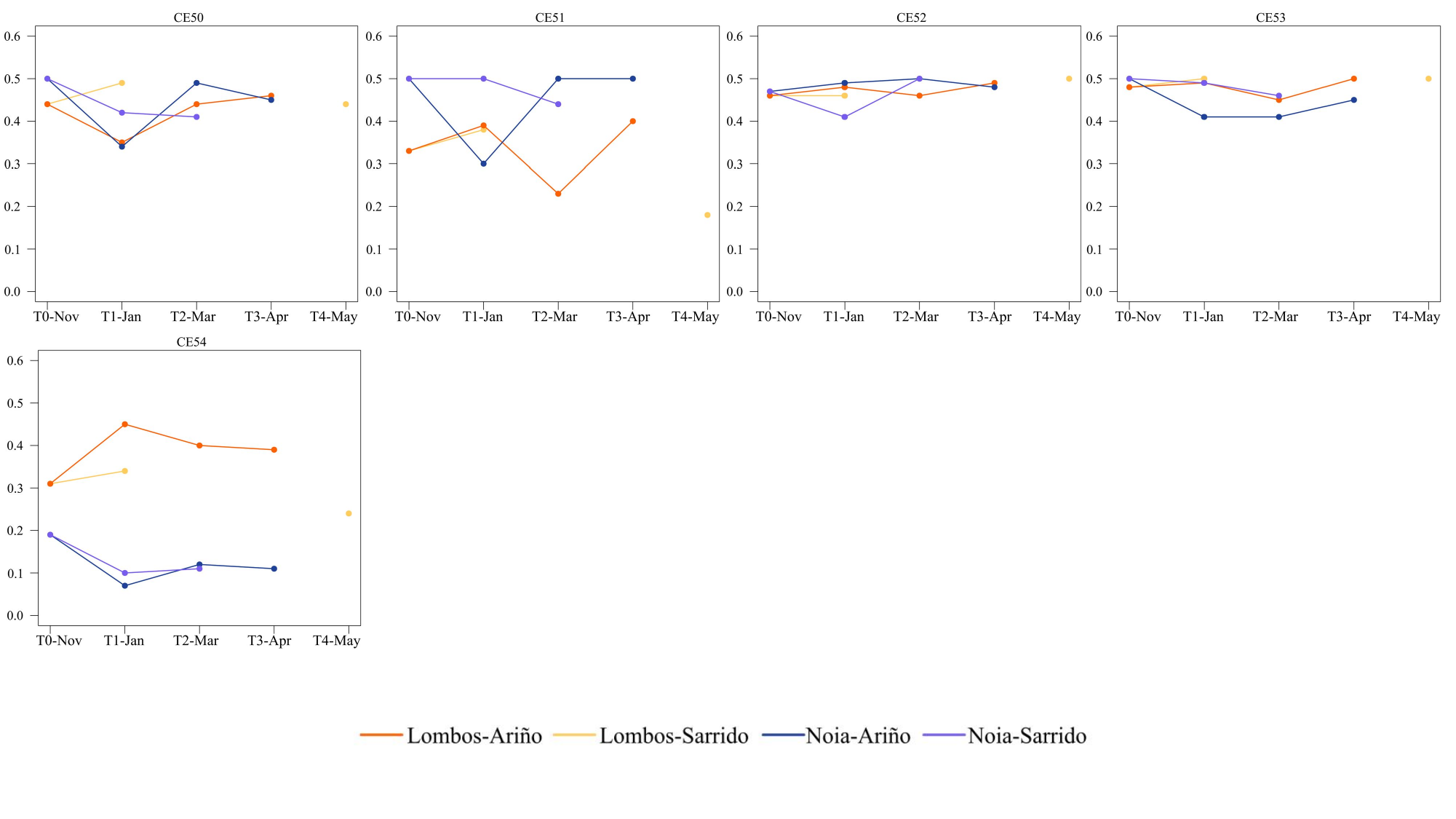

Supplement: Supplementary file 3 — Figure S3. [file EVA-16-1789-s012.pptx]

## Slide 1
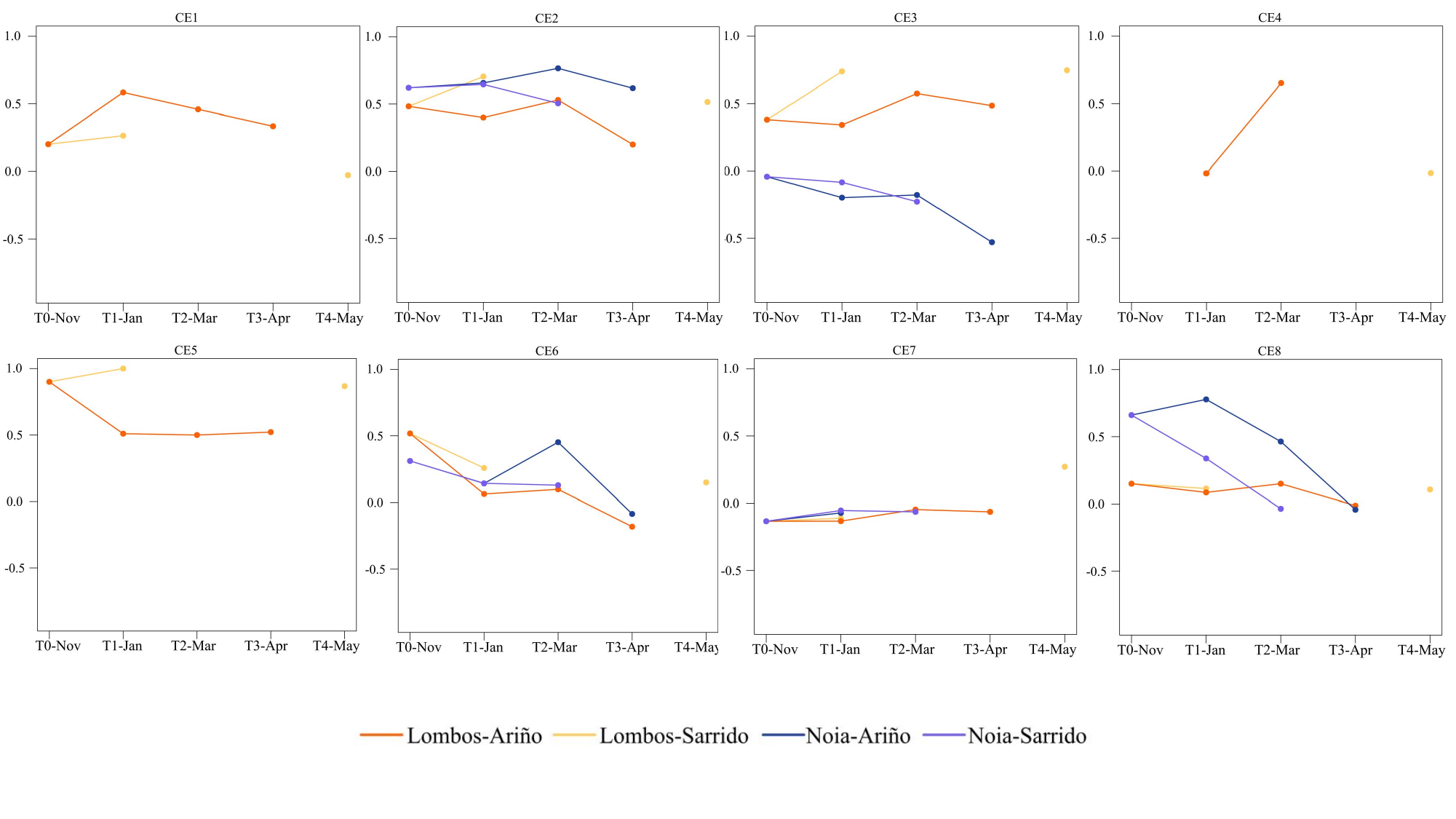

## Slide 2
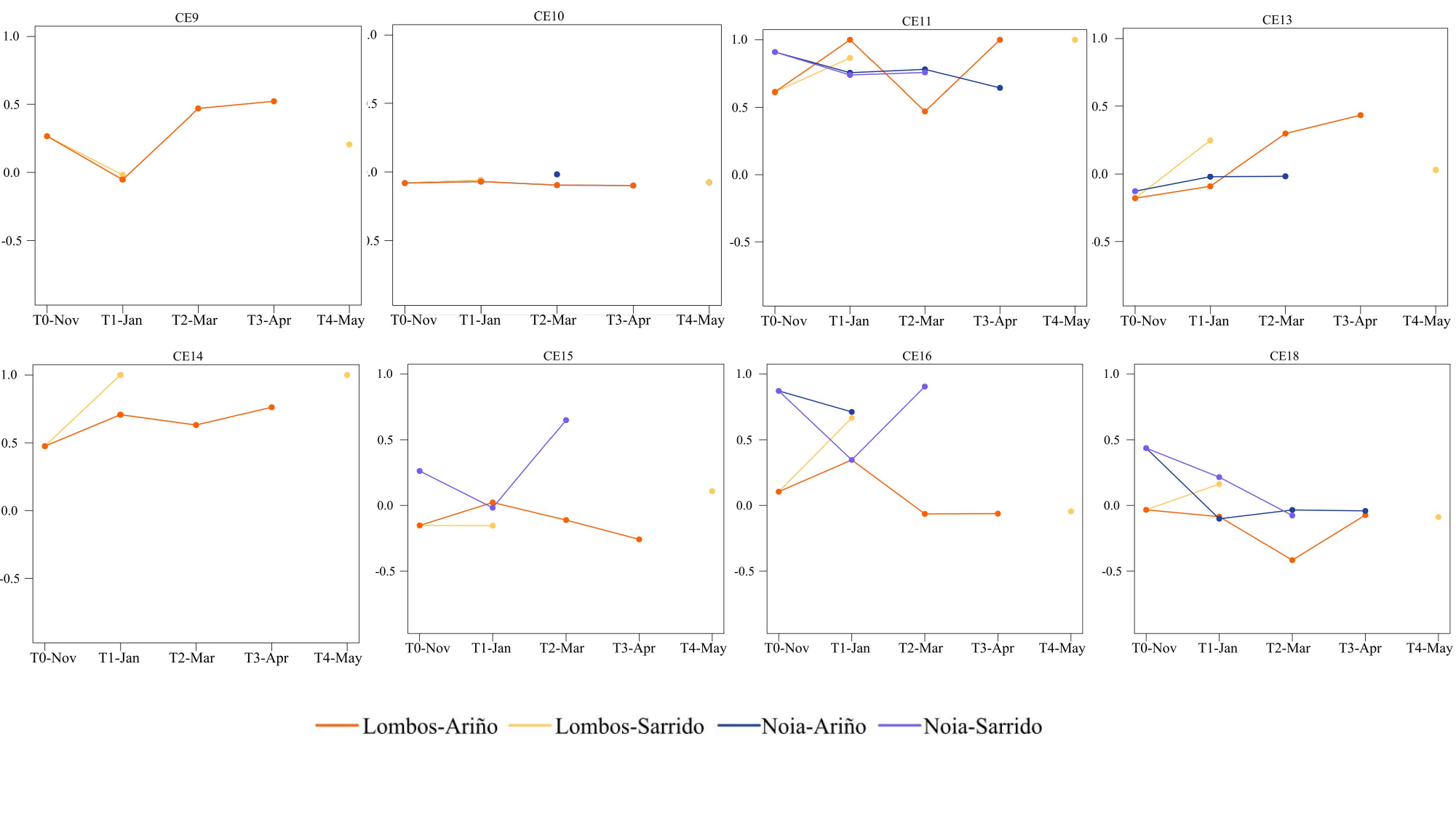

## Slide 3
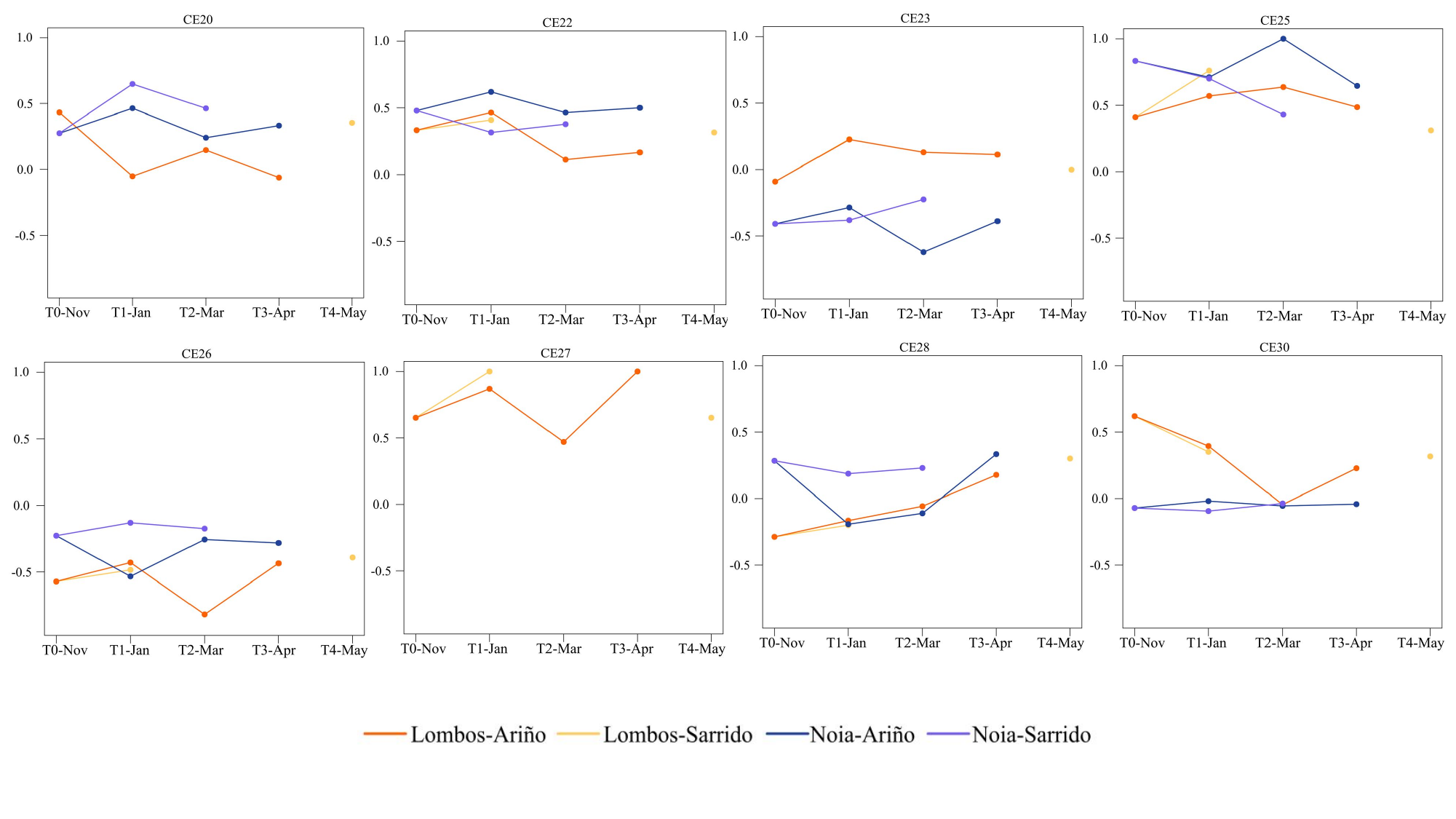

## Slide 4
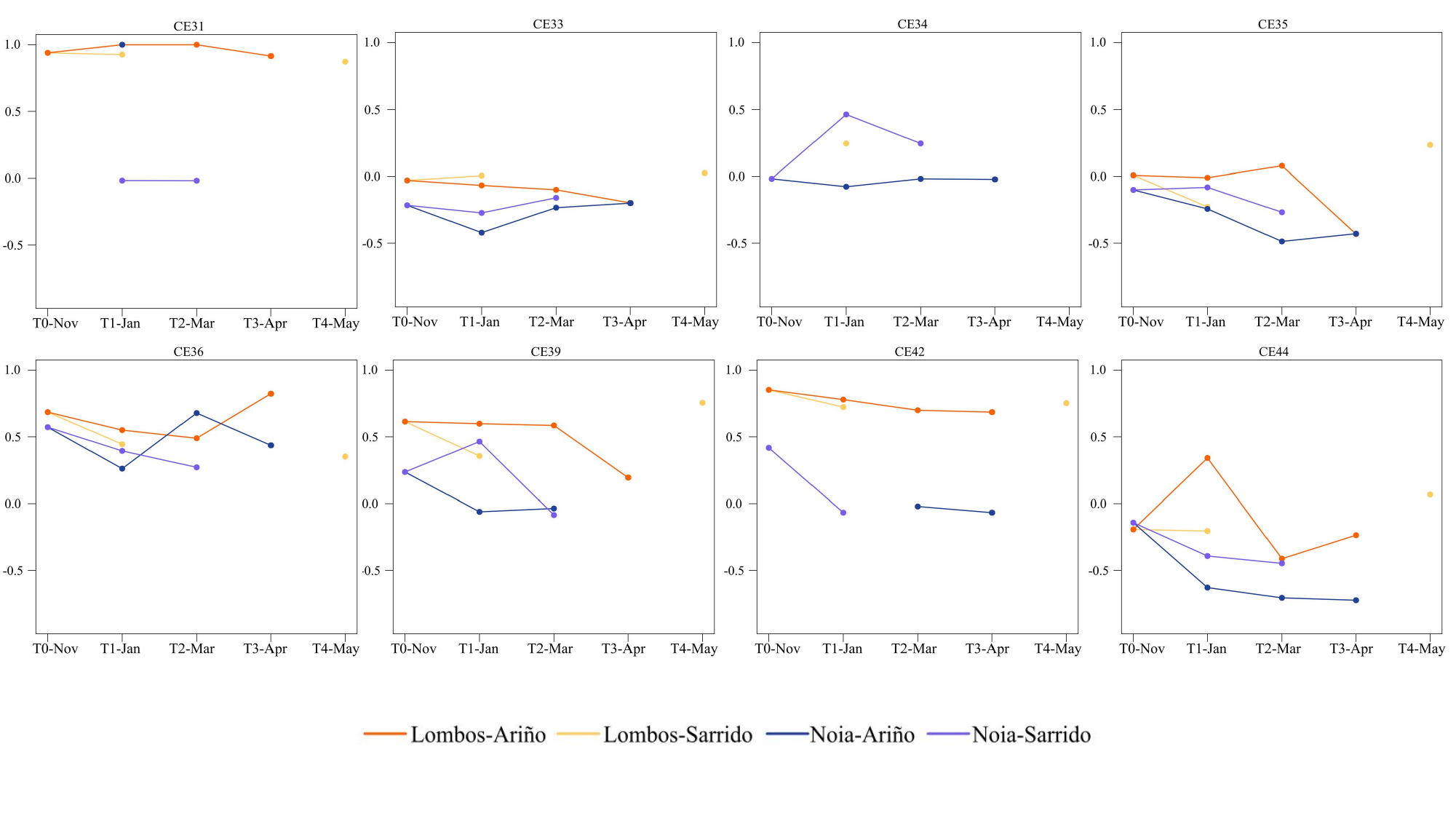

## Slide 5
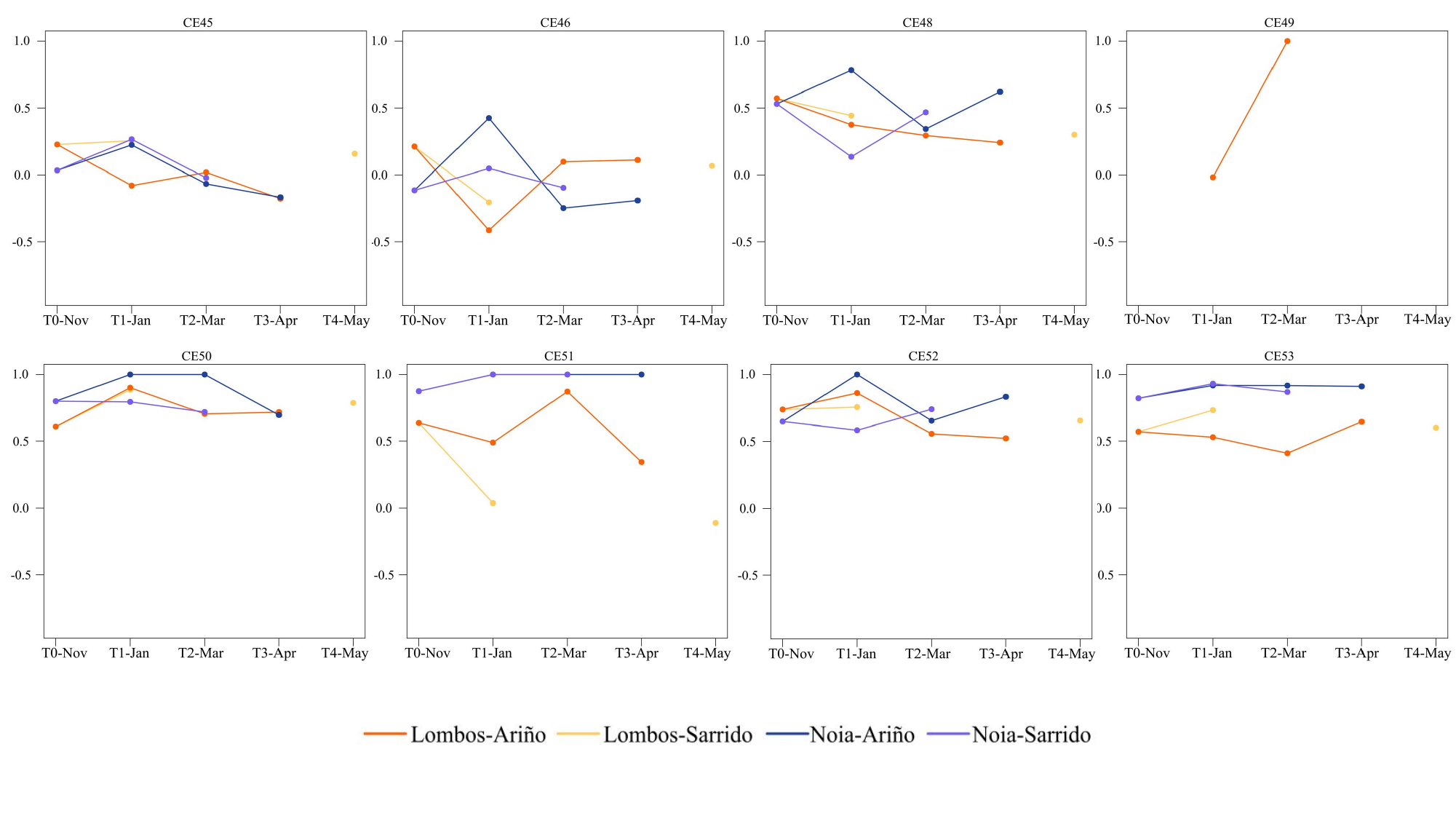

## Slide 6
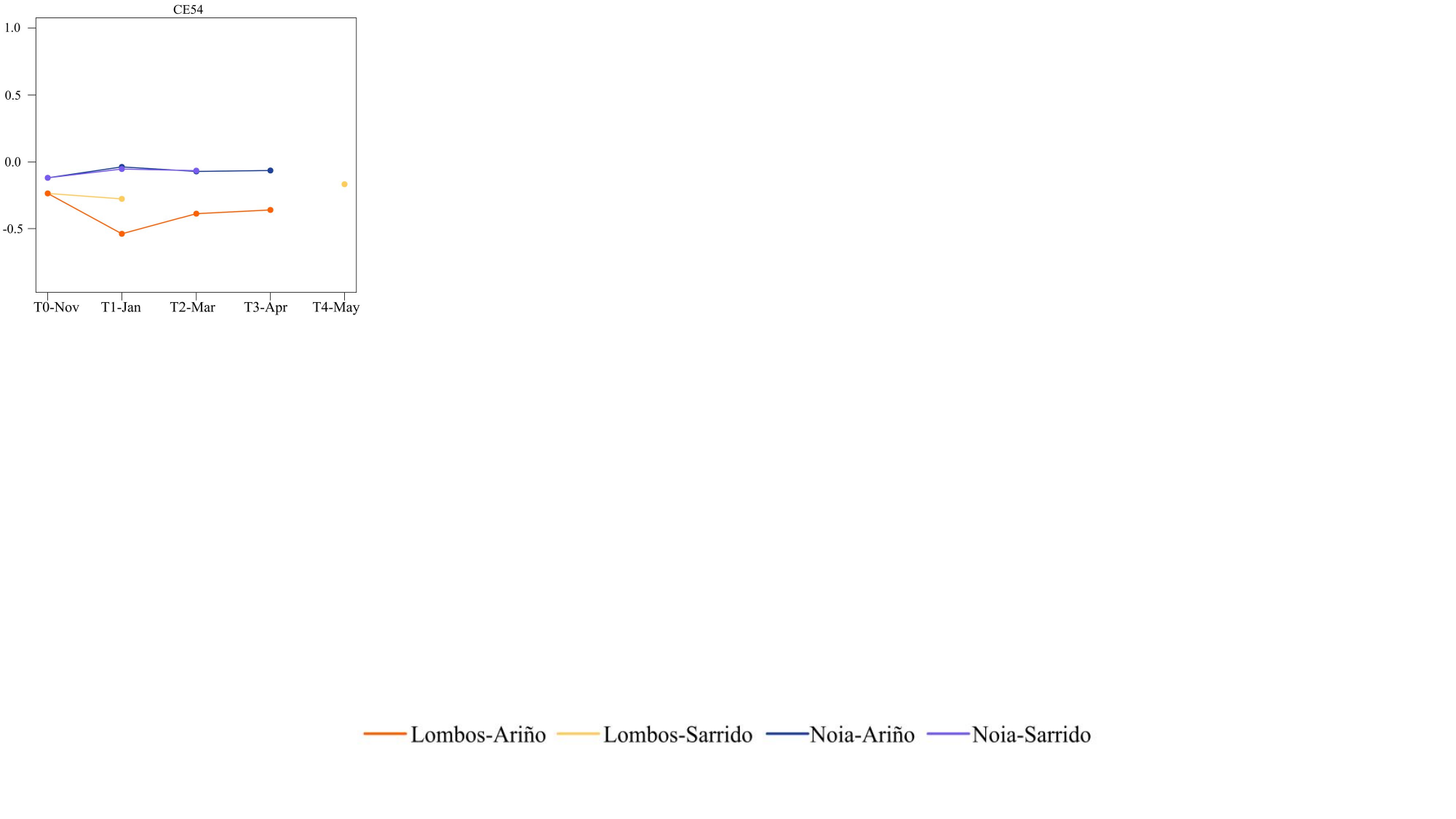

Supplement: Supplementary file 4 — Figure S4. [file EVA-16-1789-s011.pptx]

## Slide 1
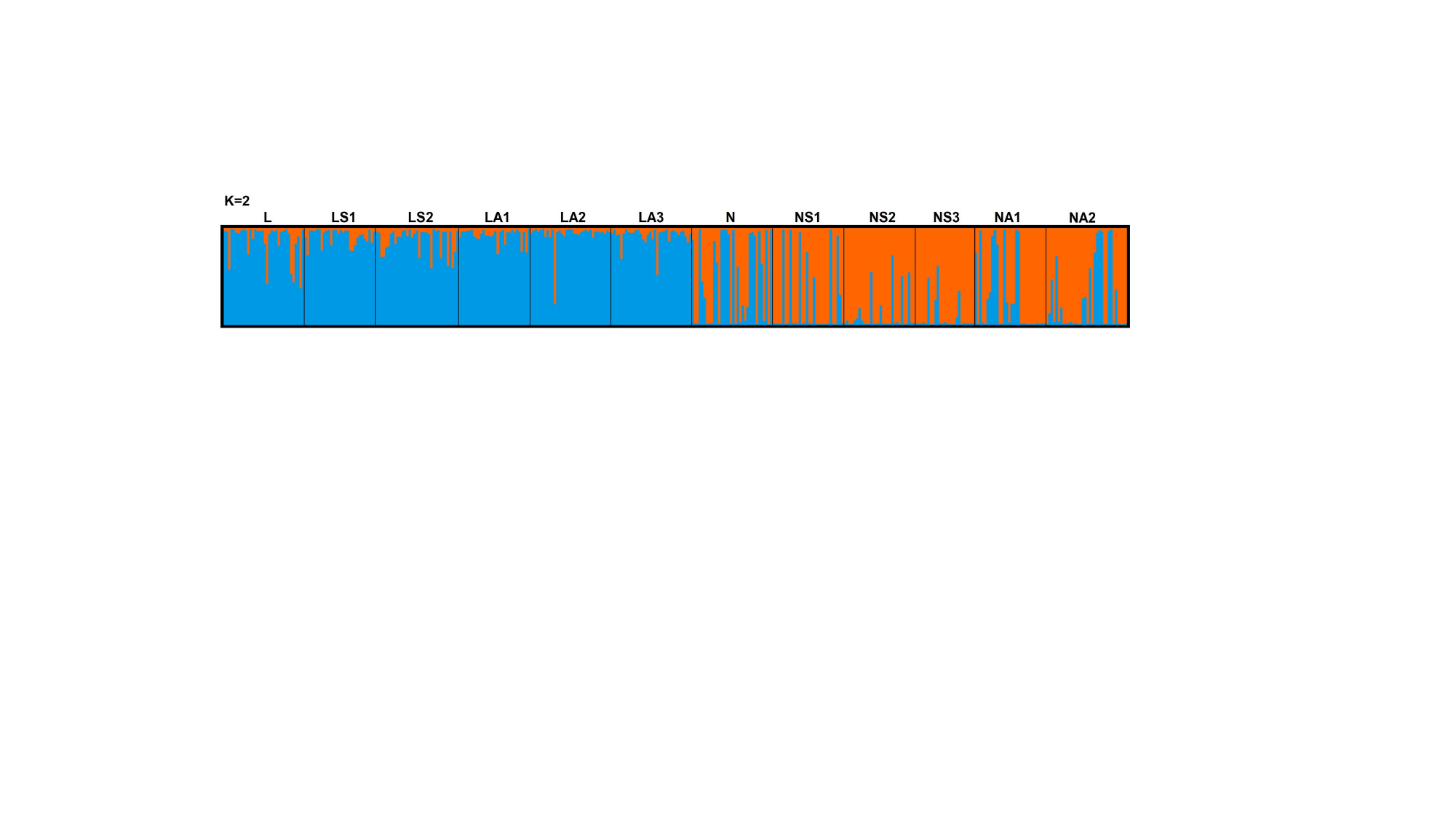

Supplement: Supplementary file 5 — Figure S5. [file EVA-16-1789-s006.pptx]

## Slide 1
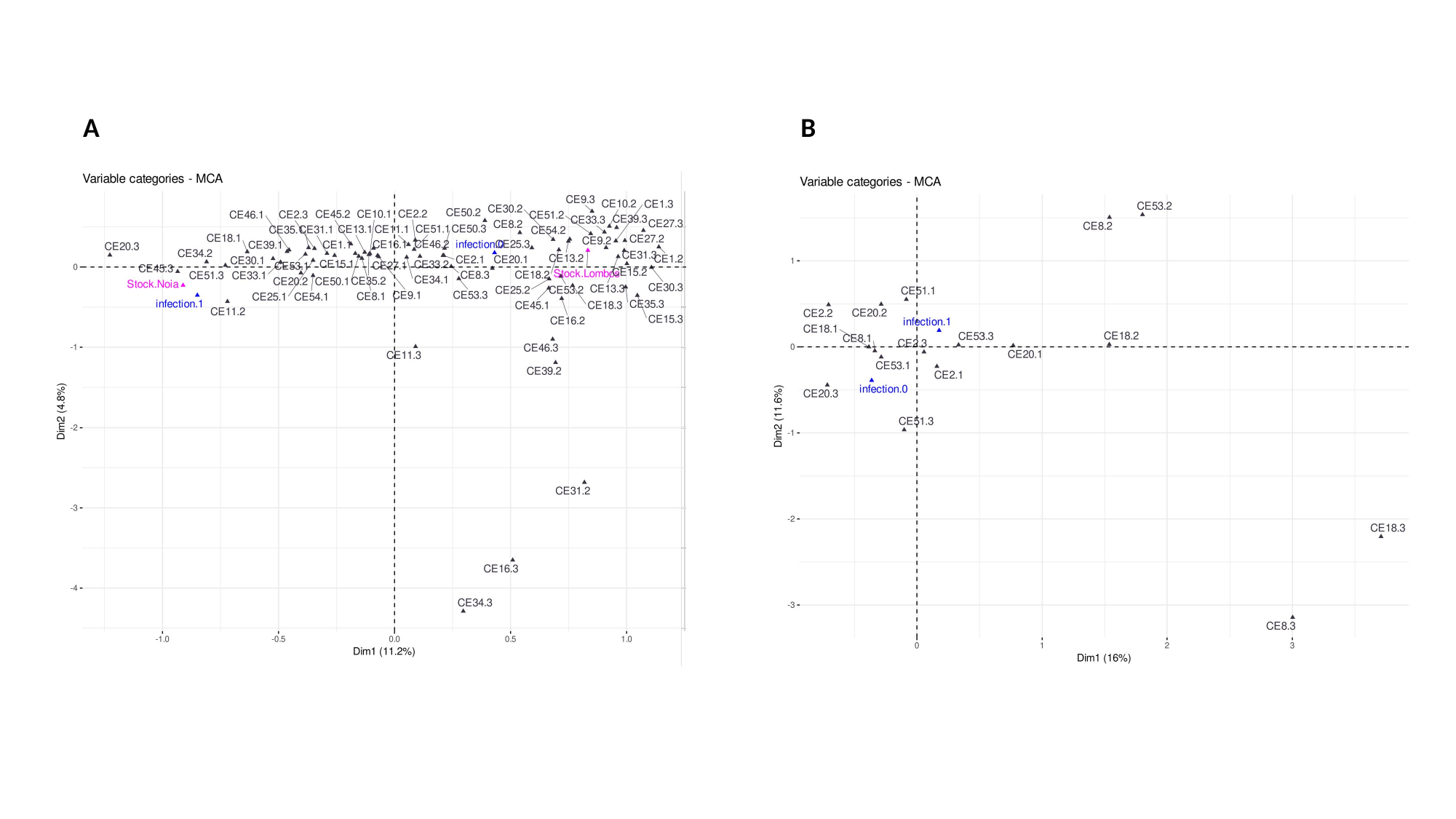

A
B

Supplement: Supplementary file 6 — Figure S6. [file EVA-16-1789-s003.pptx]
